# Acyl-Coenzyme A Synthetase Long-Chain Family Member 4 Is Involved in Viral Replication Organelle Formation and Facilitates Virus Replication via Ferroptosis

**DOI:** 10.1128/mbio.02717-21

**Published:** 2022-01-18

**Authors:** Yu-An Kung, Huan-Jung Chiang, Mei-Ling Li, Yu-Nong Gong, Hsin-Ping Chiu, Chuan-Tien Hung, Peng-Nien Huang, Sheng-Yu Huang, Pei-Yu Wang, Tsu-An Hsu, Gary Brewer, Shin-Ru Shih

**Affiliations:** a Research Center for Emerging Viral Infections, College of Medicine, Chang Gung University, Taoyuan City, Taiwan; b Department of Biochemistry & Molecular Biology, Rutgers Robert Wood Johnson Medical School, Piscataway, New Jersey, USA; c Department of Laboratory Medicine, Linkou Chang Gung Memorial Hospital, Taoyuan, Taiwan; d Division of Infectious Diseases, Department of Pediatrics, Linkou Chang Gung Memorial Hospital, Taoyuan City, Taiwan; e Institute of Biotechnology and Pharmaceutical Research, National Health Research Institutes, Miaoli County, Taiwan; f Department of Medical Biotechnology and Laboratory Science, College of Medicine, Chang Gung University, Taoyuan, Taiwan; g Research Center for Chinese Herbal Medicine, Research Center for Food and Cosmetic Safety, and Graduate Institute of Health Industry Technology, College of Human Ecology, Chang Gung University of Science and Technology, Taoyuan, Taiwan; Johns Hopkins University; Johns Hopkins Bloomberg School of Public Health

**Keywords:** genome-wide CRISPR screens, ACSL4, enterovirus, coronavirus, ferroptosis

## Abstract

Enterovirus infections can cause severe complications, such as poliomyelitis, encephalitis, myocarditis, meningitis, neurological pulmonary edema, and even death. Here, we used genome-wide CRISPR screens to gain new insight into the mechanism by which enteroviruses co-opt host pathways to potentiate replication and propagation. We found that acyl-coenzyme A synthetase long-chain family member 4 (ACSL4) is involved in viral replication organelle formation. ACSL4 is a key component of ferroptosis, an iron-dependent, nonapoptotic programmed cell death. Our results indicated that enteroviruses and coronaviruses can induce ferroptosis via ACSL4. Most importantly, ferroptosis inhibitors, including two FDA-approved drugs, rosiglitazone (ROSI; ACSL4 inhibitor) and pioglitazone (PIO; ACSL4 inhibitor), decreased the viral load of human enteroviruses and coronaviruses, suggesting that ACSL4 is a target for counteracting viral infection.

## INTRODUCTION

*Enterovirus*, a genus in the family *Picornaviridae*, includes four human enterovirus species and three human rhinovirus species. Human enteroviruses, which include polioviruses, coxsackie A and B viruses, and echoviruses, cause various diseases, such as the common cold, hand-foot-and-mouth disease (HFMD), herpangina, and acute hemorrhagic conjunctivitis. Certain enterovirus infections cause severe complications, including poliomyelitis, encephalitis, myocarditis, meningitis, neurological pulmonary edema, and death (1). The substantial number of serotypes of this family represents a challenge for the development of vaccines that can target multiple enteroviruses. Therefore, broad-spectrum antivirals remain warranted.

CRISPR-Cas9 screening is a powerful high-throughput tool for identifying common host factors critical for virus propagation (2–4). Using CRISPR-Cas9 screening, we identified acyl-coenzyme A (CoA) synthetase long-chain family member 4 (ACSL4) as a critical factor for coxsackievirus A6 (CV-A6) infection. CV-A6, which belongs to human enterovirus A, is a major cause of HFMD and herpangina. ACSL4 catalyzes polyunsaturated fatty acids (PUFAs), such as arachidonic acid (AA) and eicosapentaenoic acid (EPA), into acyl-CoA and is involved in eicosanoid biosynthesis, cancer progression, and pathways associated with mental retardation (5). ACSL4 is also essential for ferroptosis, a recently recognized form of iron-dependent cell death characterized by accumulated lipid peroxidation (6). An increase in lipid reactive oxygen species (ROS) induces ferroptosis due to the depletion of intracellular glutathione (GSH) levels and inactivation of glutathione peroxidase 4 (GPX4) (7). Ferroptosis is involved in multiple diseases, including tumors, neurodegenerative disorders, stroke, and ischemia/reperfusion injury (8). However, the influence of ACSL4 on ferroptosis during viral infections has yet to be defined.

In this study, we uncovered the roles of ACSL4 in viral replication. Moreover, we provide proof-of-concept results illustrating that enteroviruses and coronaviruses can induce ferroptosis via ACSL4. Previous studies have indicated that two FDA-approved drugs, rosiglitazone (ROSI) and pioglitazone (PIO) (trade names Avandia and Actos, respectively), can impede ferroptosis by ACSL4 inhibition. Accordingly, we further established the inhibitory effects of ROSI and PIO on the titers of various enteroviruses and coronaviruses, including SARS-CoV-2. Taken together, our results provide a new strategy to reduce enterovirus and coronavirus viral yields via an antiferroptotic mechanism by inhibiting ACSL4.

## RESULTS

### ACSL4 is a broad host factor for enterovirus replication.

**FIG 1:**
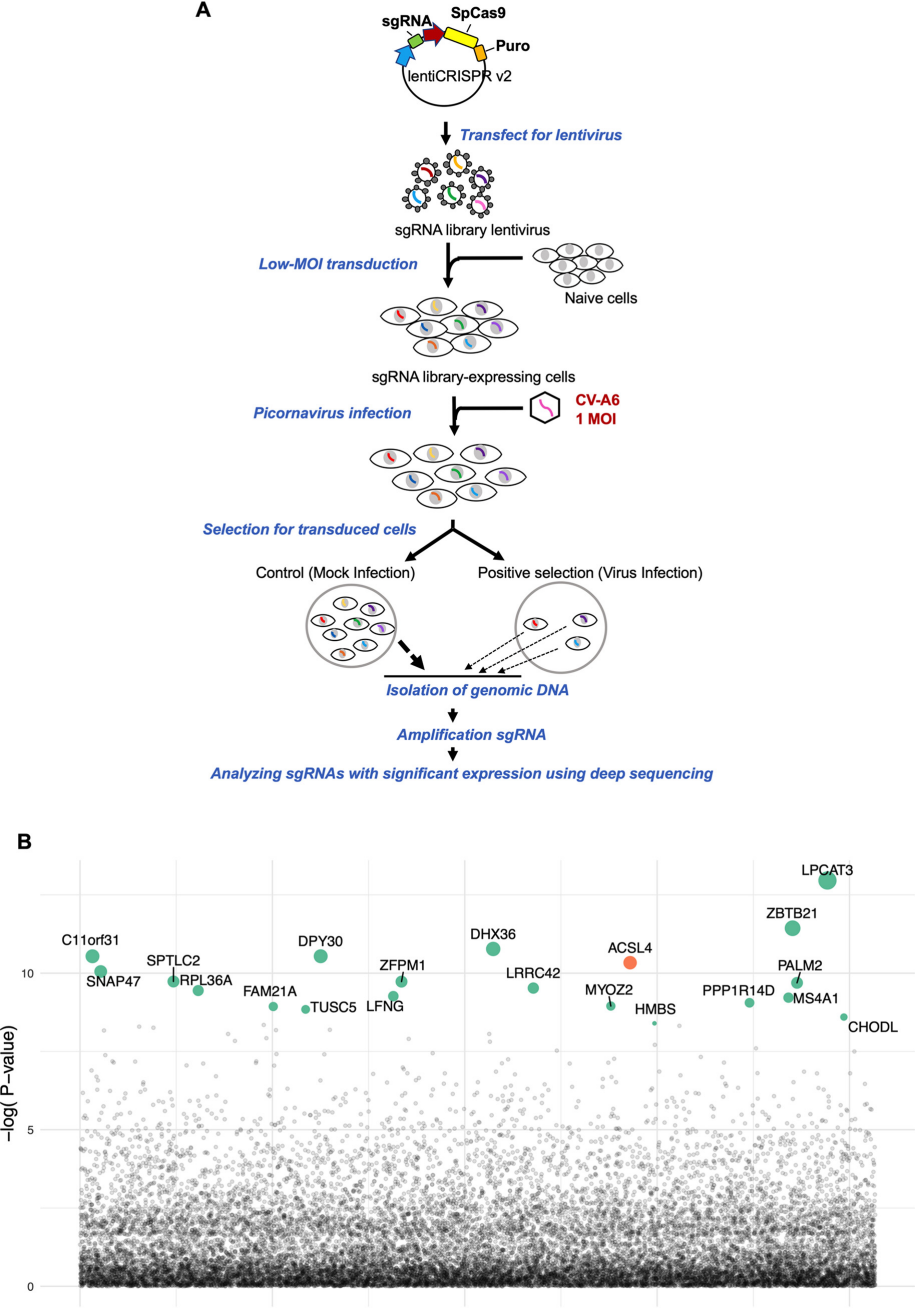
Genome-wide CRISPR-Cas9 screens for CV-A6 revealing genes involved in viral replication. (A) Flowchart of the CRISPR-Cas9 screening process for genes involved in viral replication. A genome-scale CRISPR-Cas9 knockout study involved the construction of a plasmid library containing genes encoding the effector protein and sgRNAs. Plasmids were packaged into the lentivirus vector and then transduced into the A549 cells to generate cell lines with stable expression for screening. These cell lines were infected with CV-A6 at an MOI of 1, and genomic DNA was harvested at designated time points postinfection. The sgRNA regions were amplified from the genomic DNA and analyzed by deep sequencing followed by a MAGeCK analysis to identify candidate genes. (B) Top 20 sgRNAs, including the *ACSL4* gene in orange color and others in green, identified from the established sgRNA library. The sizes of the circles indicate the magnitude of increase in sgRNA expression.

We searched for host factors involved in CV-A6 replication by CRISPR-Cas9 screening. We performed a genome-scale CRISPR-Cas9 knockout screen with CV-A6 at a multiplicity of infection (MOI) of 1 in A549 cells transduced with single guide RNAs (sgRNAs) from the GeCKO v2 human library (Fig. 1A). Genomic DNA was harvested from colonies of uninfected (mock infection) and surviving cells (virus infection). Additionally, sgRNA colonies were amplified by PCR and subjected to deep sequencing using the Illumina NextSeq platform. Model-based analysis of genome-wide CRISPR/Cas9 knockout (MAGeCK) was used to prioritize and identify significant sgRNAs from our deep sequencing data. Figure 1B presents the top 20 candidates enriched by the CV-A6 challenge, namely, ACSL4, LPCAT3, ZBTB21, DPY30, DHX36, C11orf31, ZFPM1, PALM2, SNAP47, SPTLC2, RPL36A, FAM21A, TUSC5, LFNG, LRRC42, MYOZ2, HMBS, PPP1R14D, MS4A1, and CHODL. ACSL4 and LPCAT3 are important factors in ferroptosis (9, 10); however, the contribution of ACSL4 to virus-induced ferroptosis has yet to be defined. Therefore, we focused on ACSL4 in further analyses.

**FIG 2:**
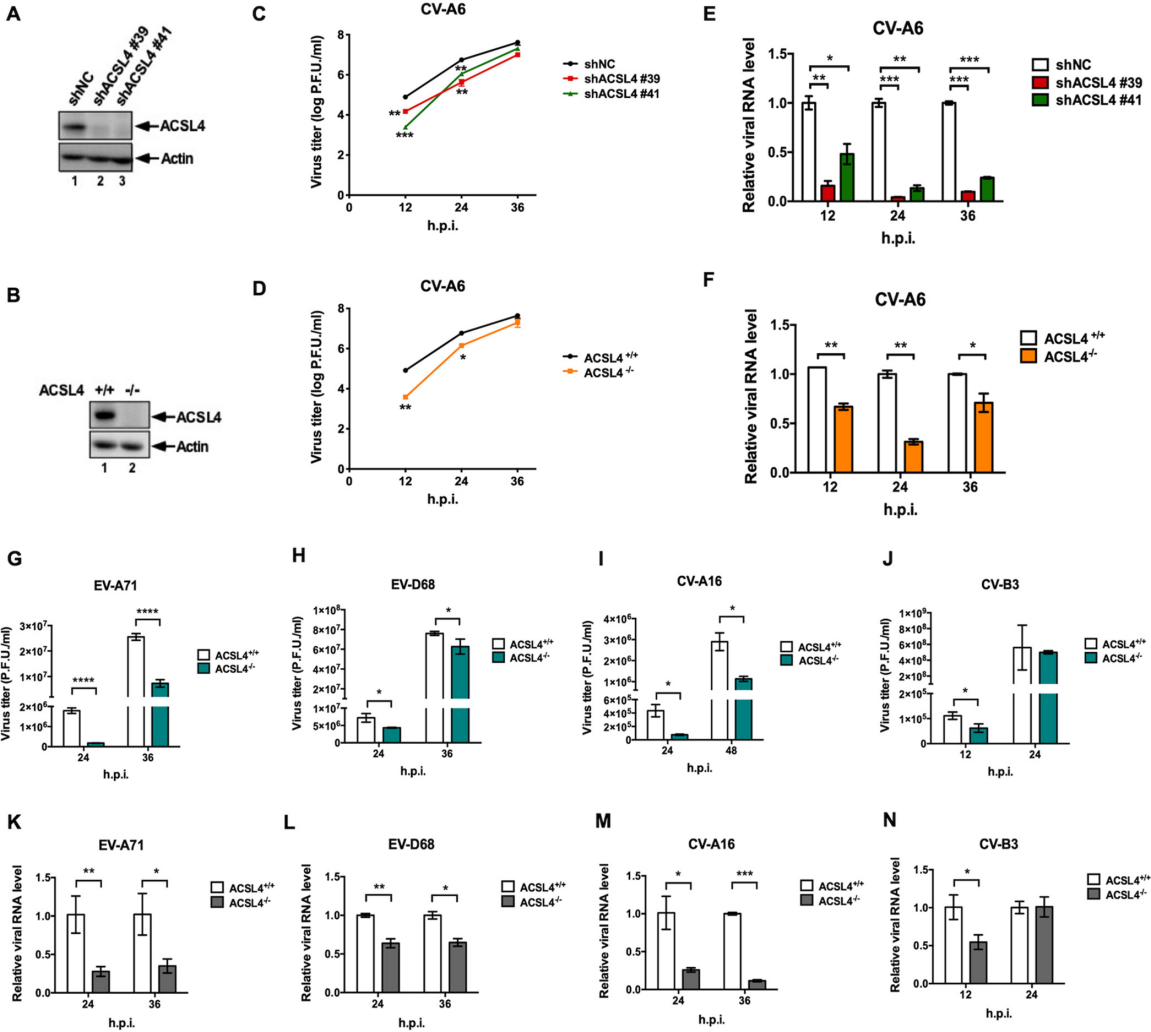
ACSL4 is a broad host factor for enterovirus replication. RD cells were used to generate ACSL4 KD and ACSL4^−/−^ cells. Western blots represent the expression of ACSL4 in ACSL4 KD cells (A) and in ACSL4^−/−^ cells (B). (C) Viral yields of CV-A6 in ACSL4 KD cells. ACSL4 KD cells (shACSL4 #39 and shACSL4 #41) and negative-control cells (shNC) were infected with CV-A6 at an MOI of 0.01. Viruses were harvested at the indicated time points, and viral titers were determined by a plaque assay. (D, G, H, I, and J) ACSL4^−/−^ and ACSL4^+/+^ cells developed using CRISPR-Cas9 editing were infected with CV-A6 at an MOI of 0.01 (D), EV-A71 at an MOI of 0.001 (G), EV-D68 at an MOI of 0.001 (H), CV-A16 at an MOI of 0.1 (I), or CV-B3 at an MOI of 0.001 (J). Viruses were harvested at the indicated time points, and viral titers were measured by a plaque assay. (E, F, K, L, M, and N) RNA quantification of enteroviruses in ACSL4 KD and ACSL4^−/−^ cells. (E) ACSL4 KD and shNC cells were infected with CV-A6 at an MOI of 0.01. ACSL4^−/−^ and ACSL4^+/+^ cells were infected with CV-A6 at an MOI of 0.01 (F), EV-A71 at an MOI of 0.001 (K), EV-D68 at an MOI of 0.001 (L), CV-A16 at an MOI of 0.1 (M), or CV-B3 at an MOI of 0.001 (N). Cells were harvested at the indicated time points. RNA extraction followed by quantitative reverse transcription PCR (qRT-PCR) was performed. Data are presented as means ± standard deviations (SD) from three independent experiments and analyzed using Student’s two-tailed unpaired *t* tests. *, *P < *0.05; **, *P < *0.01; ***, *P < *0.001; ****, *P < *0.0001.

ACSL4, a member of the ACSL family, can activate long-chain fatty acids for lipid biosynthesis and the degradation of fatty acids (11, 12). We established ACSL4 knockdown (KD) cells (shACSL4 #39 and shACSL4 #41) and ACSL4 knockout cell lines (ACSL4^−/−^) using the CRISPR-Cas9 gene-editing system to investigate the biological roles of ACSL4 in viral replication (Fig. 2A and B). The expression levels of ACSL4 were significantly lower in shACSL4 #39 and shACSL4 #41 cells (Fig. 2A, lanes 2 and 3) than in the negative control (shNC) (Fig. 2A, lane 1); the same results were obtained in a comparison of ACSL4^−/−^ cells (Fig. 2B, lane 2) and ACSL4^+/+^ cells (Fig. 2B, lane 1). Moreover, cell viability did not significantly differ between ACSL4^+/+^ and ACSL4^−/−^ cells (see also Fig. S1A in the supplemental material). To determine the viral titer of CV-A6 in ACSL4 knockdown (KD) and ACSL4^−/−^ cells, we collected CV-A6 at 12, 24, and 36 h postinfection in ACSL4 KD (Fig. 2C) or ACSL4^−/−^ cells (Fig. 2D) at an MOI of 0.01. The viral titers of CV-A6 decreased 81%, 92%, and 74% at 12, 24, and 36 h postinfection, respectively, in the shACSL4 #39 cells (Fig. 2C, red line) compared with those for shNC cells (Fig. 2C, black line). In addition, the viral titers of CV-A6 decreased drastically by 97%, 80%, and 50% at 12, 24, and 36 h postinfection in the shACSL4 #41 cells (Fig. 2C, green line) compared with levels for shNC cells (Fig. 2C, black line). In ACSL4^−/−^ cells, the viral yields of CV-A6 decreased 95%, 76%, and 55%, respectively, at 12, 24, and 36 h postinfection (Fig. 2D, orange line) compared with yields for ACSL4^+/+^ cells (Fig. 2D, black line). Moreover, the RNA expression levels of CV-A6 were significantly lower in ACSL4 KD and ACSL4^−/−^ cells than in control cells (Fig. 2E and F). To clarify the role of intact ACSL4 in mediating CV-A6 infection, ACSL4^−/−^ cells were reconstituted with wild-type ACSL4 (ACSL4^WT^) or mutants affecting the catalytic activity of ACSL4 (ACSL4^G401L^ or ACSL4^G401R^) (13) (Fig. S1B). Both ACSL4 mutants (ACSL4^G401L^ and ACSL4^G401R^) failed to rescue virus propagation in ACSL4^−/−^ cells, while the virus yields were restored in cells reconstituted with wild-type ACSL4. We also performed an ACSL4^WT^ dose-dependent experiment and found that virus titers of CV-A6 increased in a dose-dependent manner (Fig. S1C). Moreover, the mRNA and protein expression levels of endogenous ACSL4 were examined to address whether ACSL4 is activated upon CV-A6 infection. The mRNA expression level of *ACSL4* increased slightly during CV-A6 infection, especially at 36 h postinfection (Fig. S1D), while no significant changes were observed in the protein expression levels of ACSL4 during viral infection (Fig. S1E).

10.1128/mBio.02717-21.1 FIG S1 Catalytic activity of ACSL4 involved in CV-A6 replication. (A) ACSL4 gene knockout did not affect cell proliferation. Equal numbers of RD ACSL4^+/+^ and ACSL4^−/−^ cells were seeded in 96-well plates. Cell viability was measured by the CellTiter96 Aqueous One solution cell proliferation assay (Promega) at 1, 2, and 3 days postseeding. Data are presented as means ± SD from three independent experiments. Experimental data were analyzed using Student’s two-tailed unpaired *t* tests. (B) Viral yields of CV-A6 in RD ACSL4^+/+^ cells, ACSL4^−/−^ cells, and ACSL4^−/−^ cells with ACSL4 reconstitution (ACSL4^WT^, ACSL4^G401L^, or ACSL4^G401R^). RD cells were transfected with 2 μg of ACSL4^WT^, ACSL4^G401L^, or ACSL4^G401R^ plasmid constructs. After 24 h, RD cells, including ACSL4^+/+^, ACSL4^−/−^, and ACSL4 reconstitution (ACSL4^WT^, ACSL4^G401L^, or ACSL4^G401R^), were infected with CV-A6 at an MOI of 0.01 for 36 h. Viruses were harvested and the viral yields were determined by a plaque assay. Cell lysates were collected for Western blotting to examine ACSL4 expression levels with an anti-ACSL4 antibody. Actin was used as an internal control. (C) RD cells were transfected with 0.5 or 2 μg of ACSL4^WT^ plasmid for 24 h and then infected with CV-A6 at an MOI of 0.01 for 36 h. Viruses were harvested and the viral yields were determined by a plaque assay. Cell lysates were collected for Western blotting for ACSL4, CV-A6 VP1, and actin analysis. (D) RD cells were infected with CV-A6 at an MOI of 0.01. Cells were harvested at the indicated time points. RNA extraction followed by quantitative reverse transcription PCR (qRT-PCR) was performed. (E) Western blots and quantitative results for the expression of ACSL4 in CV-A6-infected cells. Data are presented as means ± SD from three independent experiments and analyzed using Student’s two-tailed unpaired *t* tests. *, *P* < 0.05; **, *P < *0.01; ***, *P < *0.001; ****, *P* < 0.0001. Download FIG S1, TIF file, 0.7 MB. Copyright © 2022 Kung et al. 2022 Kung et al. https://creativecommons.org/licenses/by/4.0/ This content is distributed under the terms of the Creative Commons Attribution 4.0 International license.

Decreases in viral titers and RNA expression levels of other enterovirus species were also observed in ACSL4^−/−^ cells, including EV-A71 (human enterovirus A) (Fig. 2G and K), EV-D68 (human enterovirus D) (Fig. 2H and L), CV-A16 (human enterovirus A) (Fig. 2I and M), and CV-B3 (human enterovirus B) (Fig. 2J and N). ACSL4 is involved in lipid metabolism; lipids are essential for membrane formation in the viral replication complex, virus assembly, and virus release (14). Therefore, we further investigated whether ACSL4 is also involved in the replication of other viral taxa, including enveloped and nonenveloped viruses. We found that ACSL4 is involved in the replication of these other RNA viruses. The viral titers of coronavirus-229E (CoV-229E), influenza A virus (IAV), and Zika virus (ZIKV) also decreased in ACSL4^−/−^ cells or ACSL4 KD cells (Fig. S2). Collectively, these findings indicate that ACSL4 is a common host factor critical for the replication of enteroviruses and other RNA viruses, including CoV, IAV, and ZIKV.

10.1128/mBio.02717-21.2 FIG S2 ACSL4 is involved in the replication of other RNA viruses. (A) RD ACSL4^−/−^ and ACSL4^+/+^ cells were infected with CoV-229E at an MOI of 0.01. Viruses were collected at 1 to 4 days postinfection, and viral titers were measured by a plaque assay. Data are presented as means ± SD from three independent experiments and analyzed using Student’s two-tailed unpaired *t* tests. *, *P < *0.05; ****, *P < *0.0001. (B and C) A549 ACSL4 KD cells (shACSL4 #39) and shNC cells were infected with IAV at an MOI of 0.001. Cell lysates and viruses were harvested at the indicated time points. Cell lysates were analyzed by Western blotting using antibodies against IAV M1, ACSL4, and actin (B), and virus titers were determined by a plaque assay (C). (D and E) ZIKV was cultured with A549 ACSL4 KD cells (shACSL4 #39) and shNC cells an MOI of 0.1. Cell lysates were analyzed by Western blotting using antibodies against ZIKV NS2B, ZIKV NS3, ACSL4, and GADPH (D), and viral titers were determined by a plaque assay (E). Download FIG S2, TIF file, 0.9 MB. Copyright © 2022 Kung et al. 2022 Kung et al. https://creativecommons.org/licenses/by/4.0/ This content is distributed under the terms of the Creative Commons Attribution 4.0 International license.

### ACSL4 affects viral replication organelle formation.

**FIG 3:**
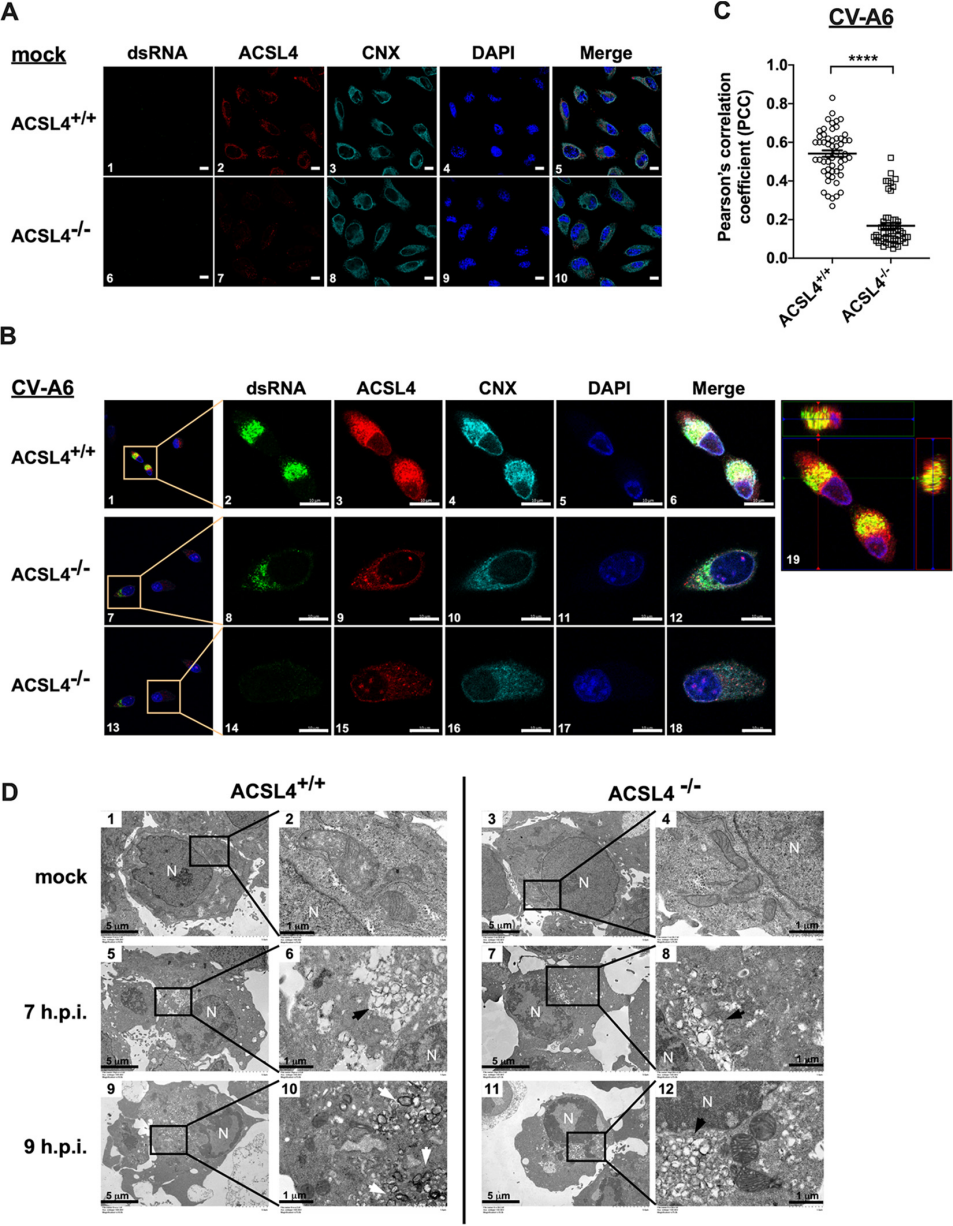
ACSL4 is involved in viral replication organelle formation. (A and B) The colocalization of dsRNA, ACSL4, and CNX in RD ACSL4^+/+^ or ACSL4^−/−^ cells. ACSL4^+/+^ and ACSL4^−/−^ cells were challenged with CV-A6 at an MOI of 20 for 7 h, fixed, stained with DAPI and antibodies against dsRNA, ACSL4, and CNX, and then examined by confocal microscopy. Scale bar, 20 μm (A) and 5 μm (B). (C) Colocalization of dsRNA and ACSL4 was estimated by Pearson’s correlation coefficients in RD ACSL4^+/+^ or ACSL4^−/−^ cells. Means ± SD were calculated from 60 cells in each group; ****, *P < *0.0001. (D) RD ACSL4^+/+^ and ACSL4^−/−^ cells were mock infected or infected with CV-A6 at an MOI of 20, and images were obtained by transmission electron microscopy at 7 and 9 h postinfection. Virus-induced vesicular clusters (black arrow) and double-membrane vesicles (DMVs) (white arrow) are shown in images 6, 8, 10, and 12. Low-magnification images are shown in images 1, 3, 5, 7, 9, and 11. High-magnification images are shown in images 2, 4, 6, 8, 10, and 12. N, nucleus. Scale bar, 5 μm in images 1, 3, 5, 7, 9, and 11 and 1 μm in images 2, 4, 6, 8, 10, and 12.

Since viral yields and RNA expression were significantly reduced in ACSL4^−/−^ cells, we investigated whether ACSL4 is involved in viral replication organelle (RO) formation. Enterovirus infection induces cellular membrane remodeling, which is involved in the recruitment of viral proteins, host proteins, and lipids (15, 16). Previous studies have indicated that enteroviruses use the endoplasmic reticulum (ER) and Golgi membranes to initiate the viral replication complex, which is an important site for viral replication (17). Double-stranded RNAs (dsRNAs) indicate viral RNA (vRNA) synthesis and are thought to be a marker for the viral replication complex (18). ACSL4 localizes in the ER, lipid droplets, mitochondria, and plasma membrane (19). Therefore, we evaluated whether ACSL4 colocalized with dsRNA and calnexin (CNX; an ER marker) to determine whether it is involved in the viral replication complex. The localization of CV-A6 dsRNA, ACSL4, and CNX was examined in mock- and CV-A6-infected ACSL4^+/+^ and ACSL4^−/−^ cells (Fig. 3A and B). ACSL4^+/+^ or ACSL4^−/−^ cells were infected with CV-A6 at an MOI of 20, and the subcellular distributions of dsRNA, ACSL4, and CNX were analyzed by fluorescence confocal microscopy at 7 h postinfection. The dsRNA signal was significantly lower in ACSL4^−/−^ cells (Fig. 3B, images 8 and 14) than in ACSL4^+/+^ cells (Fig. 3B, image 2). The merged image of dsRNA (green fluorescence) and ACSL4 (red fluorescence) in the orthoview of Z-stack images of CV-A6-infected ACSL4^+/+^ cells showed the colocalization of dsRNA with ACSL4 (Fig. 3B, image 19). Quantitative analyses based on Pearson’s correlation coefficients further revealed the colocalization of dsRNA and ACSL4 at viral replication sites in ACSL4^+/+^ cells upon CV-A6 infection (Fig. 3C).

Transmission electron microscopy (TEM) was further applied to observe the CV-A6 RO generation in ACSL4^+/+^ and ACSL4^−/−^ cells (Fig. 3D). ACSL4^+/+^ or ACSL4^−/−^ cells were infected with CV-A6 at MOI of 20. Virus-induced modifications were observed in the perinuclear region at 7 h postinfection (Fig. 3D, images 5, 6, 7, 8, black arrow) in a comparison with mock controls (Fig. 3D, images 1, 2, 3, 4). A virus-induced difference in membranous structures occupying most of the cytoplasm was observed in ACSL4^+/+^ cells at 9 h postinfection (Fig. 3D, image 9) compared with 7 h postinfection (Fig. 3D, image 5). A number of double-membrane vesicles (DMVs) were also observed in ACSL4^+/+^ cells (Fig. 3D, image 10, white arrow) but not in ACSL4^−/−^ cells (Fig. 3D, image 12) at 9 h postinfection. Therefore, CV-A6 RO formation was delayed in ACSL4^−/−^ cells (Fig. 3D, images 7, 8, 11, 12) compared to ACSL4^+/+^ cells (Fig. 3D, images 5, 6, 9, 10). Taken together, ACSL4 is involved in CV-A6 RO formation.

### Enteroviruses induce ferroptosis via ACSL4.

**FIG 4:**
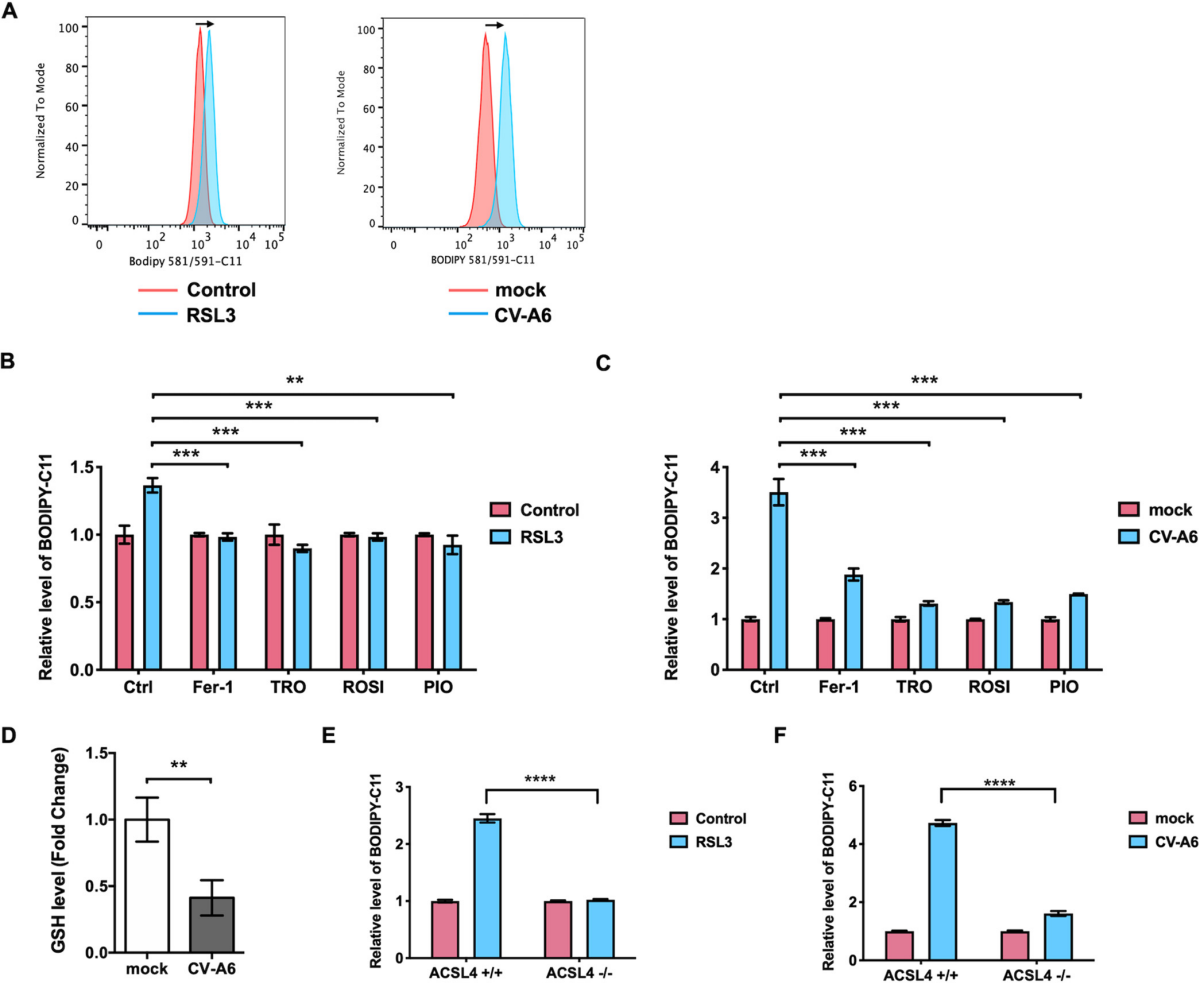
CV-A6 induces ferroptosis via ACSL4. (A) CV-A6 infection increases lipid peroxidation. RD cells were infected with CV-A6 at an MOI of 0.01 for 36 h and treated with the ferroptosis inducer RSL3 (1 μM) as a positive control. Cells were stained with 1 μM BODIPY 581/591-C11, a lipid peroxide sensor, prior to flow cytometry. The staining data obtained at 530 nm (oxidized BODIPY 581/591-C11) are plotted as a histogram. (B and C) BODIPY oxidation induced by RSL3 (B) or CV-A6 (C) with or without ferroptosis inhibitor treatment. RD cells were infected with CV-A6 at an MOI of 0.01 in the absence or presence of the ferroptosis inhibitors (Fer-1 [100 μM], TRO [20 μM], ROSI [80 μM], and PIO [40 μM]) for 36 h. RD cells were treated with RSL3 (1 μM) as a control. Cells were stained with 1 μM BODIPY 581/591-C11 for 30 min at 37°C and analyzed by flow cytometry. (D) Depleted intracellular glutathione (GSH) levels in CV-A6-infected cells. RD cells were infected with CV-A6 at an MOI of 1 for 24 h and lysed, and intracellular GSH level was measured using a commercial glutathione assay kit (Promega). (E and F) ACSL4 is the key factor for virus-induced ferroptosis. RD ACSL4^−/−^ and ACSL4^+/+^ cells were treated with RSL3 (1 μM) (E) or infected with CV-A6 at an MOI of 0.01 (F). The cells at 36 h postinfection were stained with BODIPY 581/591-C11 and analyzed by flow cytometry. Data are presented as means ± SD from three independent experiments and analyzed using Student’s two-tailed unpaired *t* tests. **, *P < *0.01; ***, *P < *0.001; ****, *P < *0.0001.

Previous studies have demonstrated that ACSL4 plays a vital role in ferroptosis (6, 20), which is an iron-dependent, nonapoptotic regulatory cell death first described in 2012 (21). It is characterized by the accumulation of lethal lipid peroxides, especially phosphatidylethanolamine-OOH (PE-OOH). The accumulation of oxidized arachidonoyl (AA)-containing PE or adrenoyl (AdA)-containing PE can induce ferroptosis, and ACSL4 is a key enzyme for the formation of AA-OOH-PE from AA or AdA-OOH-PE from AdA (9). Therefore, to decipher the association between ferroptosis and enterovirus infection, we determined whether enteroviruses can induce ferroptosis. Since lipid peroxidation is the hallmark of ferroptosis (22), a lipid peroxidation sensor, BODIPY 581/591-C11, was used to detect lipid peroxidation in virus-infected cells. Cells were treated with RSL3, an inducer of ferroptosis, as a positive control. In parallel, other groups of cells were infected with CV-A6. An increase in lipid peroxidation was prominently noted in RSL3-treated cells and CV-A6-infected cells compared with dimethyl sulfoxide (DMSO)-treated and mock-infected, control cells (Fig. 4A). However, the increases in BODIPY 581/591-C11 staining were attenuated by ferroptosis inhibitors, namely, ferrostatin-1 (Fer-1), troglitazone (TRO), ROSI, and PIO, in RSL3-treated (Fig. 4B) and CV-A6-infected cells (Fig. 4C). A previous study has shown that TRO, ROSI, and PIO can impede ferroptosis by the inhibition of ACSL4 (6). In addition, CV-A6 can deplete approximately 60% of intracellular GSH, a downstream effector of ferroptosis (Fig. 4D). Our results also indicated that supplementing cells with an exogenous source of GSH can prevent cell death (Fig. S3). Since ACSL4 is a key factor contributing to ferroptosis, lipid peroxidation was quantified in ACSL4^−/−^ cells treated with RSL3 or infected with CV-A6. In line with previous findings, RSL3 elicited ferroptosis in ACSL4^+/+^ cells; however, RSL3 was unable to induce lipid peroxidation in ACSL4^−/−^ cells (Fig. 4E). Moreover, lipid peroxidation in ACSL4^+/+^ cells was increased by approximately 4.5-fold after CV-A6 infection compared with the mock infection. Conversely, CV-A6 only induced lipid peroxidation 1.5-fold in ACSL4^−/−^ cells compared with mock infection (Fig. 4F). Lipid peroxidation was also measured in ACSL4^−/−^ cells reconstituted with ACSL4^WT^ or catalytic mutant ACSL4 (ACSL4^G401L^ and ACSL4^G401R^). Compared with that in ACSL4^−/−^ cells, an increase in lipid peroxidation was observed in ACSL4^−/−^ cells reconstituted with ACSL4^WT^, while lipid peroxidation did not change significantly in ACSL4^−/−^ cells reconstituted with ACSL4^G401L^ and ACSL4^G401R^ (Fig. S4A). Lipid peroxidation increased in a dose-dependent manner in ACSL4^−/−^ cells reconstituted with increasing amounts of ACSL4^WT^ (Fig. S4B). Moreover, shrunken mitochondria, a morphological hallmark of ferroptosis (6), were observed in ACSL4^+/+^ cells upon CV-A6 infection (Fig. S4C, image 3, red arrow) but not in mock-infected cells (Fig. S4C, images 1 and 2) or in ACSL4^−/−^ cells with virus infection (Fig. S4C, image 4). These results indicate that ACSL4 contributes to CV-A6-induced ferroptosis.

10.1128/mBio.02717-21.3 FIG S3 Supplementation with an exogenous source of glutathione rescues virus-induced cell death. RD cells were supplied with 10 mM glutathione ethyl-ester and then infected with CV-A6 at an MOI of 0.01 for 36 h. Cell viability was measured using the CellTiter96 Aqueous One solution cell proliferation assay (Promega). Data are presented as means ± SD from three independent experiments and analyzed using Student’s two-tailed unpaired *t* tests. ****, *P < *0.0001. Download FIG S3, TIF file, 0.09 MB. Copyright © 2022 Kung et al. 2022 Kung et al. https://creativecommons.org/licenses/by/4.0/ This content is distributed under the terms of the Creative Commons Attribution 4.0 International license.

10.1128/mBio.02717-21.4 FIG S4 ACSL4 contributes to virus-induced ferroptosis. (A) RD cells, including ACSL4^+/+^, ACSL4^−/−^, and ACSL4^−/−^ cells with ACSL4 reconstitution (ACSL4^WT^, ACSL4^G401L^ or ACSL4^G401R^), were infected with CV-A6 at an MOI of 0.01 for 36 h. Cells were stained with 1 μM BODIPY 581/591-C11 for 30 min at 37°C and analyzed by flow cytometry. Cell lysates were collected for Western blotting to examine ACSL4 expression levels with an anti-ACSL4 antibody. Actin was used as an internal control. (B) RD cells were transfected with 0.5 or 2 μg of ACSL4^WT^ plasmid for 24 h and then infected with CV-A6 at an MOI of 0.01 for 36 h. Cells were stained with 1 μM BODIPY 581/591-C11 for 30 min at 37°C and analyzed by flow cytometry. Cell lysates were collected for Western blotting for ACSL4, CV-A6 VP1, and actin analysis. Data are presented as means ± SD from three independent experiments and analyzed using Student’s two-tailed unpaired *t* tests. *, *P < *0.05; ****, *P < *0.0001. (C) RD ACSL4^+/+^ and ACSL4^−/−^ cells were mock infected or infected with CV-A6 at an MOI of 20, and images were obtained by transmission electron microscopy at 9 h postinfection. Shrunken mitochondria were observed in ACSL4^+/+^ cells infected with CV-A6 (red arrow) in image 3. Scale bar, 1 μm. Download FIG S4, TIF file, 1.1 MB. Copyright © 2022 Kung et al. 2022 Kung et al. https://creativecommons.org/licenses/by/4.0/ This content is distributed under the terms of the Creative Commons Attribution 4.0 International license.

**FIG 5:**
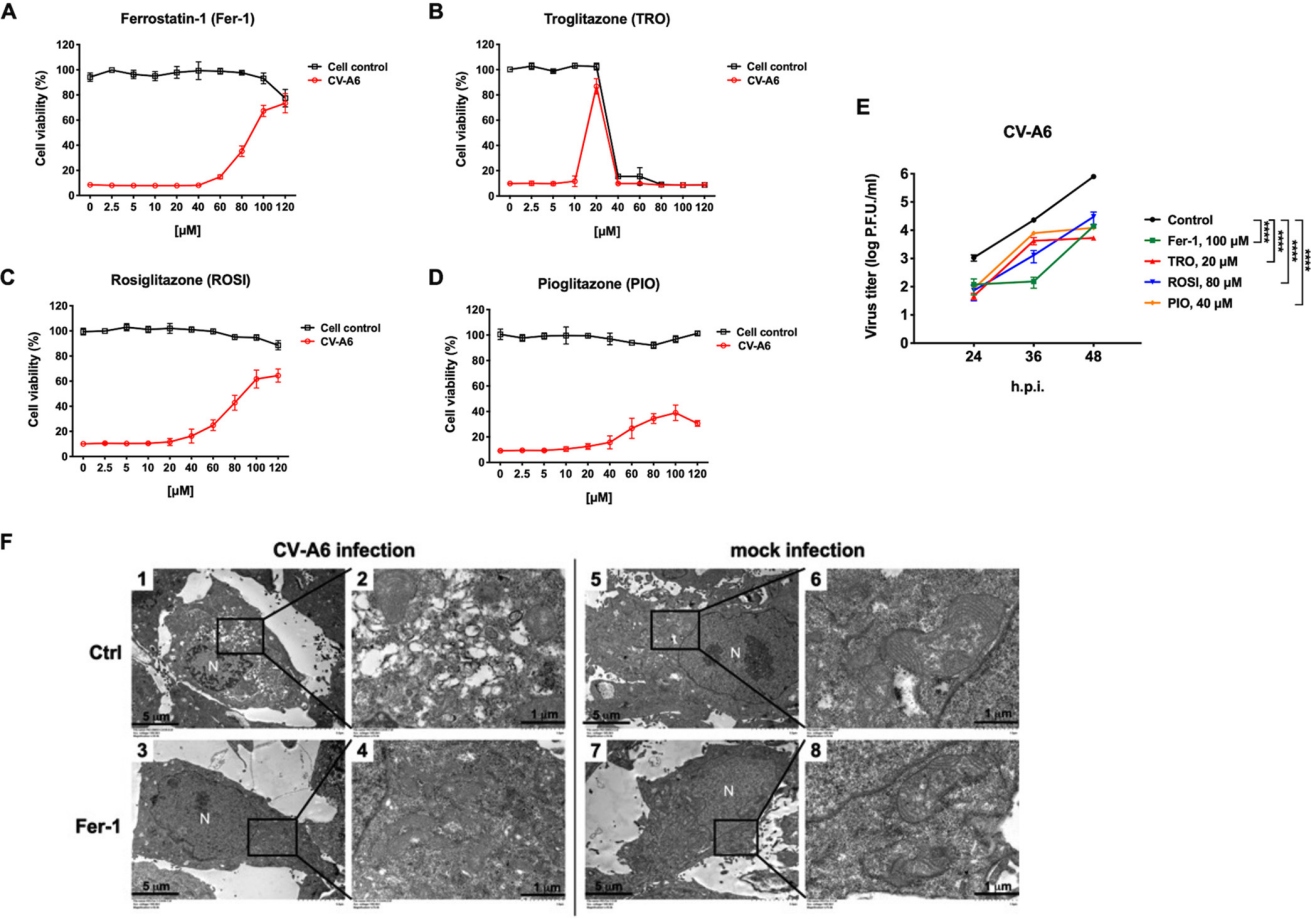
Ferroptosis inhibitors reduce CV-A6 replication. RD cells were challenged with CV-A6 in the presence of increasing concentrations of the ferroptosis inhibitor Fer-1 (A), TRO (B), ROSI (C), or PIO (D) for 36 h. Mock-infected cells were used as a control. Cell viability was measured using the CellTiter96 Aqueous One solution cell proliferation assay (Promega). Data are presented as means ± SD from three wells of a 96-well plate. (E) Ferroptosis inhibitors (Fer-1, TRO, ROSI, and PIO) reduced viral titers of CV-A6. RD cells were treated with Fer-1 (100 μM) (green line), TRO (20 μM) (red line), ROSI (80 μM) (blue line), or PIO (40 μM) (orange line) and infected with CV-A6 at an MOI of 0.01. Viruses were collected at 24, 36, and 48 h postinfection, and viral titers were measured using a plaque assay. Data are presented as means ± SD from three independent experiments: ****, *P < *0.0001. Statistical significance was determined by two-way ANOVA. (F) RD were mock-infected or infected with CV-A6 at an MOI of 20 in the presence of Fer-1 (100 μM), and images were obtained by transmission electron microscopy at 7 h postinfection. Low-magnification images are shown in images 1, 3, 5, and 7. High-magnification images are shown in images 2, 4, 6, and 8. N, nucleus. Scale bar: 5 μm in images 1, 3, 5, and 7 and 1 μm in images 2, 4, 6, and 8.

We also quantified the viability of virus-infected cells treated with the ferroptosis inhibitors Fer-1, TRO, ROSI, and PIO by the 3-(4,5-dimethylthiazol-2-yl)-5-(3-carboxymethoxyphenyl)-2-(4-sulfophenyl)-2H-tetrazolium cell proliferation assay and found that all four agents can rescue the viability of virus-infected cells (Fig. 5A to D, red line). Viral growth curves of CV-A6 were generated for cells treated with the ferroptosis inhibitors Fer-1, TRO, ROSI, and PIO. Based on the viability of inhibitor-treated mock- and virus-infected cells (Fig. 5A to D), Fer-1, TRO, ROSI, and PIO were used at concentrations of 100, 20, 80, and 40 μM, respectively. The concentrations of inhibitors were determined based on a lack of cytotoxicity and can recuse the viability of CV-A6-infected cells. These results indicated that ferroptosis and ACSL4 inhibitors could significantly inhibit viral replication (Fig. 5E). To better understand whether ferroptosis inhibitors affect the formation of viral ROs, RD cells were infected with CV-A6 at an MOI of 20 in the presence of 100 μM Fer-1 and then observed by transmission electron microscopy (TEM). The formation of CV-A6 ROs was observed in control cells at 7 h postinfection (Fig. 5F, images 1 and 2) but not in the cells treated with Fer-1 (Fig. 5F, images 3 and 4). Next, the effects of various concentrations of Fer-1 on CV-A6-induced BODIPY oxidation were evaluated. Virus-induced BODIPY oxidation decreased significantly when RD cells were treated with 80 or 100 μM Fer-1 (Fig. S5A). Moreover, CV-A6-induced cell death was rescued in the presence of 80 or 100 μM Fer-1 (Fig. S5B). The results suggest that lipid peroxidation is related to virus-induced ferroptosis.

10.1128/mBio.02717-21.5 FIG S5 Lipid peroxidation is related to virus-induced ferroptosis. RD cells were infected with CV-A6 at an MOI of 0.01 in the presence of increasing concentrations of Fer-1 for 36 h. (A) Cells were stained with 1 μM BODIPY 581/591-C11 and analyzed by flow cytometry. (B) Cell viability was measured using the CellTiter96 Aqueous One solution cell proliferation assay (Promega). Data are presented as means ± SD from three independent experiments and analyzed using Student’s two-tailed unpaired *t* tests. ****, *P < *0.0001. Download FIG S5, TIF file, 0.2 MB. Copyright © 2022 Kung et al. 2022 Kung et al. https://creativecommons.org/licenses/by/4.0/ This content is distributed under the terms of the Creative Commons Attribution 4.0 International license.

ROSI and PIO are FDA-approved drugs for the treatment of type II diabetes (23, 24). Their half-maximal inhibitory concentrations (IC_50_) against enteroviruses were determined. The IC_50_ values of ROSI against enteroviruses including CV-A6, EV-A71, EV-D68, CV-A16, and CV-B3 ranged from 15 to 27 μM (Fig. S6A). Compared with those for ROSI, PIO showed lower IC_50_ values against enteroviruses, ranging from 5 to 15 μM (Fig. S6B). Overall, our results suggest that ACSL4 influences enterovirus replication and is critical for virus-induced ferroptosis.

10.1128/mBio.02717-21.6 FIG S6 IC_50_ values of ROSI and PIO on enteroviruses. RD cells were infected with CV-A6, EV-A71, EV-D68, CV-A16, or CV-B3 at 50 PFU for the different doses of ROSI (A) or PIO (B). The viral titers were determined by a plaque reduction assay. Cytotoxicity of ROSI or PIO on uninfected RD cells was measured by the CellTiter96 Aqueous One solution cell proliferation assay (Promega). The left-hand *y* axis indicates the percentage inhibition of viral plaque formation. The right-hand *y* axis indicates the cytotoxicity of the compounds on various cell lines. The *x* axis indicates the amount of compound. IC_50_ and CC_50_ values were determined by dose-response nonlinear regression using GraphPad Prism 8. IC_50_, half-maximal inhibitory concentration; CC_50_, half-maximal cytotoxic concentration; SI, selectivity index. Download FIG S6, TIF file, 0.9 MB. Copyright © 2022 Kung et al. 2022 Kung et al. https://creativecommons.org/licenses/by/4.0/ This content is distributed under the terms of the Creative Commons Attribution 4.0 International license.

### ROSI and PIO show inhibitory effects on coronavirus replication.

**FIG 6:**
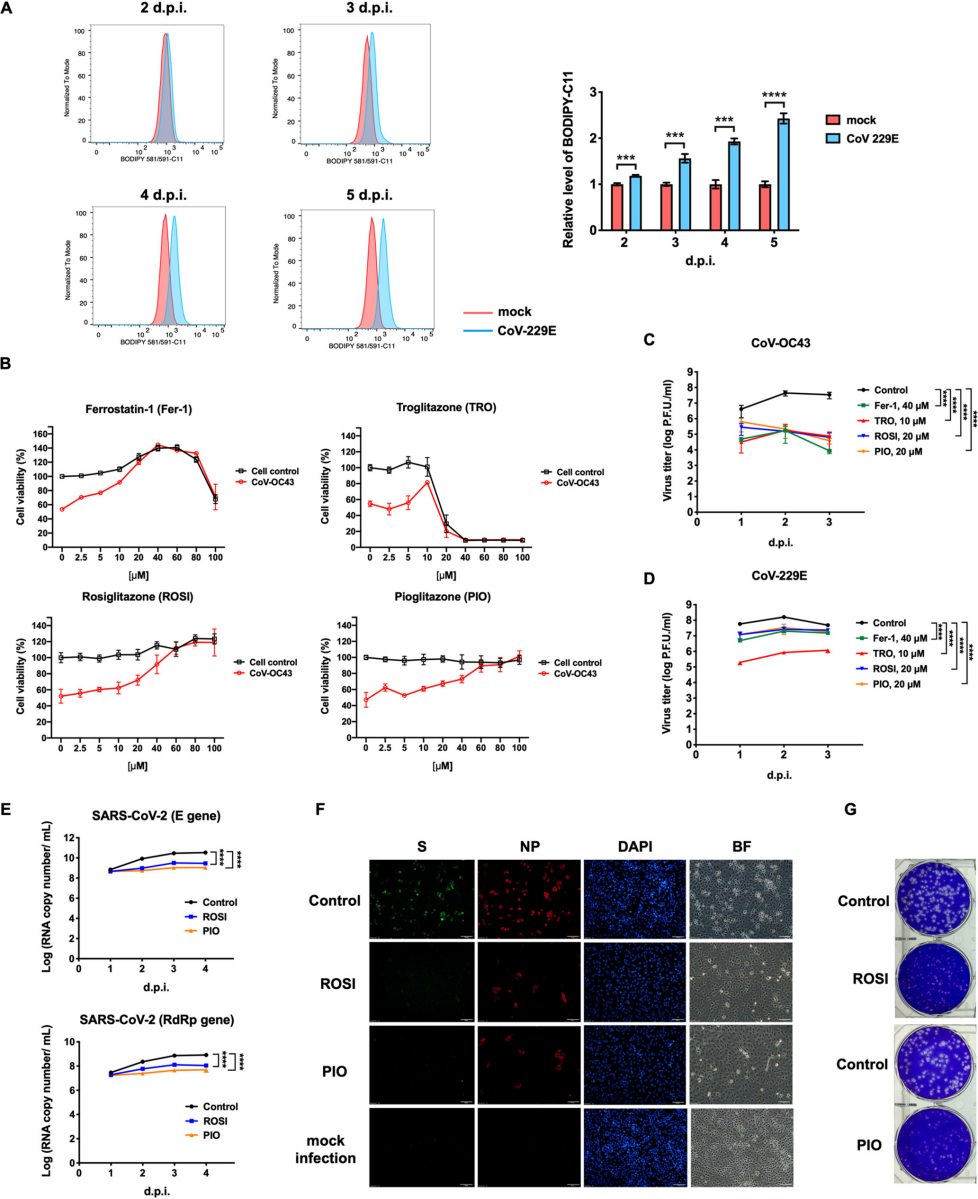
Ferroptosis inhibitors reduce coronavirus replication. (A) CoV-229E infection increased lipid peroxidation. LLC-MK2 cells were infected with CoV-229E at an MOI of 0.01 for 2 to 5 days. Cells were stained with 1 μM BODIPY 581/591-C11, a lipid peroxide sensor, prior to flow cytometry. (Left) Staining data obtained at 530 nm (oxidized BODIPY 581/591-C11) are plotted as a histogram. The relative levels of BODIPY 581/591-C11 are shown on the right. Data are representative of three independent experiments and analyzed using Student’s two-tailed unpaired *t* tests. ***, *P < *0.001; ****, *P < *0.0001. (B) Vero E6 cells were challenged with CoV-OC43 at an MOI of 0.01 in the presence of increasing concentrations of ferroptosis inhibitors Fer-1, TRO, ROSI, or PIO for 7 days. Control indicates mock-infected cells. Cell viability was measured using the CellTiter96 Aqueous One solution cell proliferation assay (Promega). Data are presented as means ± SD from three wells of a 96-well plate. (C and D) Ferroptosis inhibitors (Fer-1, TRO, ROSI, and PIO) reduced viral titers of CoV-OC43 (C) and CoV-229E (D). Vero E6 cells and LLC-MK2 cells were pretreated with Fer-1 (40 μM), TRO (10 μM), ROSI (20 μM), or PIO (20 μM) for 1 h, Vero E6 cells were infected with CoV-OC43 at an MOI of 0.01, and LLC-MK2 cells were infected with CoV-229E at an MOI of 0.01. Viruses were collected at 1, 2, and 3 days postinfection, and viral titers were measured by a plaque assay. Data are presented as means ± SD from three independent experiments: ****, *P < *0.0001. The data were analyzed by two-way ANOVA. (E) RNA quantification of SARS-CoV-2 in ROSI- or PIO-treated cells. LLC-MK2 cells were pretreated with ROSI (20 μM) or PIO (20 μM) for 1 h and then infected with SARS-CoV-2 at an MOI of 0.01 for 1 to 4 days. Viral RNA was extracted and analyzed for SARS-CoV-2 E and RdRp genes using qRT-PCR. Data are presented as means ± SD from three independent experiments: ****, *P < *0.0001. Statistical significance was determined by two-way ANOVA. (F) The expression of spike (S) and nucleocapsid (NP) of SARS-CoV-2 in ROSI- or PIO-treated cells. LLC-MK2 cells were pretreated with ROSI (20 μM) or PIO (20 μM) for 1 h, mock infected, or infected with SARS-CoV-2 at an MOI of 0.01 for 4 days, fixed, stained with DAPI and antibodies against spike and nucleocapsid proteins of SARS-CoV-2, and then examined using a fluorescence microscope. BF, bright field. Scale bar, 50 μm. (G) Plaque reduction assay of ROSI and PIO against SARS-CoV-2. Vero E6 were pretreated with ROSI (20 μM) or PIO (20 μM) for 1 h and then infected with SARS-CoV-2 for 1 h at 37°C. After virus adsorption, medium containing ROSI (20 μM) or PIO (20 μM) was added to the cell monolayer. Cells were incubated at 37°C for 3 days, fixed with 10% formaldehyde, and stained with crystal violet.

In addition to enteroviruses, the need for therapeutic strategies that are effective against coronaviruses has become a pressing issue, particularly in the context of the ongoing COVID-19 pandemic, caused by severe acute respiratory syndrome coronavirus 2 (SARS-CoV-2). Seven known coronaviruses (CoVs) infect humans: CoV-229E, CoV-NL63, CoV-OC43, CoV-HKU1, SARS-CoV, Middle East respiratory syndrome coronavirus (MERS-CoV), and SARS-CoV-2. Most coronaviruses cause mild respiratory illness; however, SARS-CoV, MERS-CoV, and SARS-CoV-2 are highly pathogenic and cause severe diseases and pandemics (25, 26). As shown in Fig. S2A, the viral titers of CoV-229E were lower in ACSL4^−/−^ cells at 1, 2, and 3 days postinfection than in ACSL4^+/+^ cells, suggesting that ACSL4 is also involved in CoV-229E replication. To determine whether coronaviruses can also induce ferroptosis, the lipid peroxidation sensor BODIPY 581/591-C11 was utilized to detect lipid peroxidation in cells infected with CoV-229E. As summarized in Fig. 6A, the coronavirus could induce ferroptosis. The viability of CoV-OC43-infected cells cultured with increasing concentrations of ferroptosis inhibitors was measured (Fig. 6B). Fer-1, TRO, ROSI, and PIO rescued the viability of CoV-OC43-infected cells at high concentrations (Fig. 6B, red line). Furthermore, the effects of ferroptosis inhibitors on viral yields of the coronavirus were also determined. Concentrations of Fer-1 (40 μM), TRO (10 μM), ROSI (20 μM), and PIO (20 μM) with no cytotoxicity against Vero E6 cells were used to determine the effects of inhibitors on CoV-OC43 yields. Viral yields of CoV-OC43 (Fig. 6C) and CoV-229E (Fig. 6D) decreased substantially in the presence of Fer-1, TRO, ROSI, and PIO. We further determined IC_50_ values of ROSI and PIO against coronaviruses, including CoV-229E and CoV-OC43. The IC_50_ values of ROSI against CoV-229E and CoV-OC43 were 11.88 and 7.817 μM, respectively (Fig. S7A). Moreover, the IC_50_ values of PIO against CoV-229E and CoV-OC43 were 10.15 and 5.67 μM, respectively (Fig. S7B). Based on the results shown in Fig. 6C and D, we examined the antiviral effects of ROSI and PIO on SARS-CoV-2. LLC-MK2 cells were pretreated with ROSI and PIO at 20 μM, followed by SARS-CoV-2 infection at an MOI of 0.01. Viral RNA was extracted at 1 to 4 days postinfection, and expression was determined using quantitative reverse transcription PCR (qRT-PCR). ROSI (Fig. 6E, blue line) and PIO (Fig. 6E, orange line) led to 1.5- to 30-fold reductions of RNA expression of the envelope (E) and RNA-dependent RNA polymerase (RdRp) genes in SARS-CoV-2 compared to levels in the control group (Fig. 6E, black line). Next, the effects of ROSI and PIO on SARS-CoV-2 infection were assessed by immunofluorescence microscopy and a plaque reduction assay. The inhibitory effects on SARS-CoV-2 infection could be readily observed in the presence of ROSI or PIO at 20 μM compared with the DMSO control (Fig. 6F). Moreover, the plaque size and the number of SARS-CoV-2 particles were lower in ROSI- or PIO-treated cells compared to those in control cells (Fig. 6G). We then compared the IC_50_ values of ROSI and PIO on SARS-CoV-2 with that of remdesivir, which is thought to be a promising antiviral agent against SARS-CoV-2 (27). LLC-MK2 cells were treated with different amounts of ROSI, PIO, or remdesivir and then infected with SARS-CoV-2 at an MOI of 0.01 for 3 days. Viral RNA was extracted and SARS-CoV-2 RdRp and E gene expression was evaluated using qRT-PCR. The IC_50_ values of ROSI and PIO against SARS-CoV-2 were comparable to the IC_50_ values of remdesivir (Fig. S7C). Taken together, the results shown in Fig. 6 provide a strong body of evidence that coronavirus can induce ferroptosis. Moreover, the ferroptosis inhibitors Fer-1, TRO, ROSI, and PIO can efficiently inhibit coronavirus replication.

10.1128/mBio.02717-21.7 FIG S7 IC_50_ values of ROSI and PIO on coronaviruses. (A and B) LLC-MK2 cells and Vero E6 cells were pretreated with various doses of ROSI (A) or PIO (B) for 1 h. LLC-MK2 cells were infected with CoV-229E at 50 PFU, and Vero E6 cells were infected with CoV-OC43 at 50 PFU for 3 days. Plaque formation units of CoV-229E or CoV-OC43 were determined by a plaque reduction assay. (C) LLC-MK2 cells were challenged with SARS-CoV-2 in the presence of increasing concentrations of remdesivir, ROSI, or PIO for 3 days. Viral RNA was extracted and analyzed for SARS-CoV-2 E and RdRp genes using qRT-PCR. Cytotoxicity of remdesivir, ROSI, or PIO on uninfected LLC-MK2 cells was measured by the CellTiter96 Aqueous One solution cell proliferation assay (Promega). IC_50_ and CC_50_ values were determined by dose-response nonlinear regression using GraphPad Prism 8. IC_50_, half-maximal inhibitory concentration; CC_50_, half-maximal cytotoxic concentration; SI, selectivity index. Download FIG S7, TIF file, 0.9 MB. Copyright © 2022 Kung et al. 2022 Kung et al. https://creativecommons.org/licenses/by/4.0/ This content is distributed under the terms of the Creative Commons Attribution 4.0 International license.

## DISCUSSION

Our results indicate that ACSL4 is a common host factor in RNA virus replication. ACSL4 is also essential for lipid metabolism, which is important in the maintenance of membrane phospholipids. Membrane rearrangement, including ER or mitochondrial membranes, is critical for viral RO formation for replication (28). Moreover, ACSL4 is localized in the ER, mitochondria, and inner side of the plasma membrane (19). By confocal imaging and electron microscopy, we found that ACSL4 may participate in CV-A6 RO formation. In addition to RO formation, viral particle assembly and release also require the host cell membrane. Although picornavirus, a nonenveloped virus, typically lyses host cells for the release of virus particles, recent evidence has indicated that picornavirus could also leave cells by a nonlytic release mechanism (i.e., naked virus virion enclosed in lipid membrane-enclosed particles called extracellular vesicles) (1). Enveloped viruses, such as influenza A virus and coronavirus, generally require orchestration between viral components on the host membrane to form an external envelope (29, 30). Therefore, we suggest that ACSL4 is involved not only in RO formation but also in virus assembly and release.

Viruses can induce cell death at different stages of infection, including viral attachment, entry, activation by viral nucleic acids, or viral protein expression. Although cell death is thought to be a strategy for host defense against virus infection (31), mounting evidence indicates that it is beneficial for the release and spread of viruses. Our results indicate that the catalytic activity of ACSL4 is essential for viral replication and virus-induced ferroptosis. Moreover, the mRNA expression level of *ACSL4* increased slightly upon CV-A6 infection, with no change in ACSL4 protein expression. Previous studies have shown that enterovirus 2A protease cleaves eukaryotic initiation factor 4G (eIF4G) to shut off cap-dependent translation, causing the protein expression levels of most host factors to decrease during enterovirus infection (32). The sustained expression level of ACSL4 indicates that it plays an essential role in virus production, since most of the host translation is shut off upon enterovirus infection. Various observations indicated that viruses can induce ferroptosis, including the accumulation of lipid peroxidation, GSH depletion, and shrunken mitochondria in virus-infected cells. GPX4 protein expression was decreased during CV-A6 infection, while both the cell susceptibility to virus and viral yields were decreased in response to ferroptosis inhibitors. Furthermore, lipid peroxidation and virus-induced cell death were suppressed at the same concentration of Fer-1. Therefore, we suggest that ferroptosis promotes virus release. This hypothesis is supported by previous results showing that cell death, such as apoptosis, can influence enterovirus release (33). However, we also observed that Fer-1 can inhibit the formation of viral ROs. Fer-1 can slow the rate of lipid peroxidation (34, 35). Previous studies have indicated that EV-A71 induces mitochondrial ROS generation, which is essential for viral replication (36). Moreover, the reduction of ROS inhibits EV-A71 replication (37). Although Fer-1 does not inhibit mitochondrial ROS production (35), lipid peroxidation may be required for CV-A6 replication. These previous findings and our results suggest that viruses recruit ACSL4 for RO formation and promote lipid peroxidation for viral replication. However, excessive lipid peroxidation, GSH depletion, and a decrease of GPX4 protein expression led to ferroptotic cell death and the release of the virus.

Previous reports have demonstrated that a variety of cell death mechanisms, including apoptosis, necroptosis, and pyroptosis, are induced in virus-infected cells (38). In addition to ferroptosis, we investigated markers of other forms of cell death upon CV-A6 infection (see Fig. S8 in the supplemental material). The hallmark of apoptosis, cleavage of poly(ADP-ribose) polymerase (PARP), was increased at 36 h postinfection, suggesting that apoptosis contributes to CV-A6-induced cell death (Fig. S8A). However, the addition of the pancaspase inhibitor Q-VD-OPh showed no effects on viral protein synthesis (Fig. S8B). These results are consistent with our previous finding that PARP is cleaved in EV-A71-infected cells; moreover, the viral protein of EV-A71 is not affected by the addition of Q-VD-OPh (39, 40). As previously reported (41), increases in receptor-interacting protein kinase 3 (RIPK3) and phosphorylation of RIPK3 were detected in CV-A6-infected cells, while mixed-lineage kinase domain-like protein (MLKL) was not phosphorylated. These results imply that the induction of necroptosis during CV-A6 infection is independent of MLKL phosphorylation (Fig. S8A). This result was further confirmed using a necroptosis inhibitor, necrostatin-1 (Nec-1), as we observed the attenuation of viral VP1 in the presence of Nec-1 (Fig. S8C). Lastly, cleavage of gasdermin D (GSDMD) and capsase-1 was observed in CV-A6-infected cells, indicating that pyroptosis was induced (Fig. S8A). The pyroptosis inhibitor disulfiram (C-23) also inhibited viral VP1 of CV-A6 (Fig. S8D). Consistent with our results shown in Fig. 5, the ferroptosis inhibitor Fer-1 had a profound effect on viral protein synthesis (Fig. S8E). Our findings establish a previously unknown role of ferroptosis in mediating viral infection. The exact mechanisms by which these viruses manipulate apoptosis, necroptosis, pyroptosis, and ferroptosis at different stages of the viral life cycle warrant further investigation.

10.1128/mBio.02717-21.8 FIG S8 CV-A6 induces apoptosis, necroptosis, and ferroptosis. (A) RD cells were infected with CV-A6 at an MOI of 0.01, and cell lysates were collected at 12, 24, and 36 h postinfection. Cell lysates were analyzed by Western blotting using antibodies against PARP, cleaved PARP, MLKL, phospho-MLKL, RIPK3, phospho-RIPK3, capspase-1, GSDMD, CV-A6 VP1, and actin. (B to E) RD cells were infected with CV-A6 at an MOI of 0.01 in the presence of Q-VD-OPh (20 μM), Nec-1 (150 μM), C-23 (10 μM), and Fer-1 (100 μM) for 36 h. Cell lysates were analyzed by Western blotting using antibodies against CV-A6 VP1, and actin. Download FIG S8, TIF file, 1.2 MB. Copyright © 2022 Kung et al. 2022 Kung et al. https://creativecommons.org/licenses/by/4.0/ This content is distributed under the terms of the Creative Commons Attribution 4.0 International license.

Our data suggest that ferroptosis inhibitors inhibited CV-A6 replication, which is particularly relevant since there are no antivirals specific for enterovirus infection. Our findings may also be pertinent to SARS-CoV-2, particularly in light of the urgent need for novel agents for the treatment of COVID-19. We found that coronaviruses can also induce ferroptosis; moreover, ferroptosis inhibitors can inhibit virus-induced cell death and viral titers of CoV-OC43 and -229E. Yang and Lai proposed that the association between SARS-CoV-2 and ferroptosis is a target for COVID-19 treatment (42). Therefore, we further examined the antiviral effects of the FDA-approved drugs ROSI and PIO on SARS-CoV-2 and found that they can decrease the RNA copy number and plaque number of SARS-CoV-2 in cell culture experiments, suggesting their potential value as antivirals for COVID-19. Taken together, the data indicate that virus can induce ferroptosis via ACSL4 and the depletion of ACSL4 can decrease viral yields by suppressing the ferroptosis mechanism. We also observed reduced RNA replication and delayed viral RO formation, both of which were attributed to ACSL4 depletion. These findings suggest that ACSL4 also participates in viral RO formation, which is essential for viral RNA synthesis. Our results suggest that ACSL4 has multiple roles in viral infection and is a potential target for antiviral therapy.

## MATERIALS AND METHODS

### Cell culture and virus infection.

Human muscle rhabdomyosarcoma (RD), embryonic kidney (293T), and lung adenocarcinoma epithelial (A549) cells were cultured at 37°C in Dulbecco’s modified Eagle’s medium (DMEM; Gibco, Waltham, MA, USA) containing 10% fetal bovine serum (FBS; Gibco). Rhesus monkey kidney epithelial cells (LLC-MK2) and African green monkey kidney (Vero-E6) cells were cultured at 37°C or 33°C in minimum essential medium (MEM; Gibco) containing 10% FBS (Gibco). Cells at 80 to 90% confluence were challenged with CV-A6 (2009-96014), CV-A16 (2010-96057), and CV-B3 (obtained from Chang Gung Memorial Hospital), EV-A71 (Tainan/4643/98), EV-D68 (TW-02795-2014), influenza A virus (A/WSN/1933), Zika virus (PRVABC59), CoV-229E (ATCC VR-740™), CoV-OC43 (ATCC VR1558), or SARS-CoV-2 (CGMH-CGU-01) at varied MOIs. After 1 h of adsorption at 37°C or 33°C in serum-free DMEM or MEM, cells were washed with phosphate-buffered saline (PBS) and incubated with DMEM or MEM containing 2% FBS.

### Lipid peroxidation with BODIPY 581/591-C11 and flow cytometry.

Cells were seeded in 12-well plates with 1 × 10^5^ cells per well. After 24 h, cells were treated with 1 μM RSL3 (Sigma, St. Louis, MO, USA) or infected with CV-A6 at an MOI of 0.01 for 48 h. Cells were incubated with 1 μM BODIPY 581/591-C11 (Invitrogen, Waltham, MA, USA) at 37°C for 30 min before collection by trypsinization. After centrifugation at 300 × *g* for 10 min, cells were resuspended in 500 μl of fresh PBS and analyzed using an Invitrogen Attune NxT flow cytometer. At least 10,000 cells per sample were analyzed, and FlowJo was used for data analysis.

### CRISPR-Cas9 genome-wide screening.

A pool of lentiviruses containing each sgRNA from the GeCKO v2 human library was obtained from the RNAi Core Lab of Academia Sinica. The system was designed by Feng Zhang (Broad Institute of MIT and Harvard, Cambridge, MA, USA). The GeCKO v2 libraries target the 5′ conserved coding exons of 19,050 human protein-coding genes, with six sgRNAs per gene and 1,864 human miRNAs with four sgRNAs per miRNA. A549 cells were transduced with the pooled lentiviral libraries at an MOI of <0.3. The low MOI ensures that most cells receive only one stably integrated RNA guide, with scaled-up transduction such that the sgRNA library has a coverage of >500 cells expressing each sgRNA. Two days after infection, cells were subjected to puromycin selection for 7 days to achieve maximal knockout efficiency, after which they were ready for screening. Pools of mutagenized cells were infected with CV-A6 at an MOI of 1. In addition, nontransduced cells were infected to ensure virus-induced cell death; transduced, uninfected cells were used as controls (mock infection). Genomic DNA (gDNA) was harvested from virally infected colonies that survived and mock-infected cells using the Blood & Cell Culture DNA kit (Qiagen, Hilden, Germany). sgRNA was amplified using PCR, and amplicons were screened for sgRNA library distribution using the NextSeq platform.

Paired-end reads were merged using USEARCH v10.0.240 (43). Reads were trimmed to remove adaptors and primers using Cutadapt v1.16 (44). MAGeCK v0.5.6 (45) was further used to prioritize and identify significant sgRNAs from sequencing data by following the tutorial (https://sourceforge.net/p/mageck/wiki/Home/).

### Cell viability assay.

Cells were seeded in a 96-well plate at 2,000 cells/well. After 24 h, cells were treated with Fer-1, TRO, ROSI, or PIO (Merck, Darmstadt, Germany). Subsequently, cells were infected with CV-A6 or CoV-OC43 at an MOI of 0.01. Cell viability was assayed at different time points after infection, and viability of infected and noninfected cells was determined using the CellTiter96 Aqueous One solution cell proliferation assay (Promega, Madison, WI, USA).

### Establishment of ACSL4 knockdown and knockout cells.

ACSL4 short hairpin RNAs (shRNAs) (TRCN0000045539 and TRCN0000045541) were purchased from the Taiwan National RNAi Core Facility, Academia Sinica. The lentivirus vector pLKO_TRC005, carrying shRNA (5′-AATTTGCGCCCGCTTACCCAGTT-3′) as the scramble control, was constructed according to the instructions of the Taiwan National RNAi Core Facility, Academia Sinica. LentiCRISPRv2 was used to create knockout cells. The ACSL4 guide RNA sequence (5′-AGGAAAGTTGTACTTAAAGC-3′) was cloned into the lentiCRISPRv2 vector following the lentiCRISPRv2 and lentiGuide-Puro protocols, with lentiviral CRISPR/Cas9 and sgRNA provided by the Feng Zhang laboratory (Broad Institute of MIT and Harvard, Cambridge, MA, USA). For lentivirus preparation, 293T cells were cotransfected with ACSL4 shRNA, LKO_TRC005-shRNA, or lentiCRISPRv2-ACSL4 sgRNA and the helper plasmids pMD.G and pCMVΔR8.91, using XtremeGENE transfection reagent (Roche, Basel, Switzerland). The culture supernatant containing viral particles was collected. RD cells or A549 cells were transduced with shACSL4 or sgRNA lentivirus for 24 h and subjected to selection with puromycin (5 μg/ml).

### Plasmids and constructs.

The coding region of ACSL4_42-711_ was cloned from RD cells (forward, 5′-AAGCTTGCGGCCGCGATGGCAAAGAGAATAAAAG-3′; reverse, 5′-GGTGGTGGTACCTTATTTGCCCCCATACATTC-3′) and then inserted into the pFLAG-CMV2 vector at NotI and KpnI enzyme sites. This plasmid subsequently served as the template for the construction of the pFLAG-CMV2-ACSL4_42-711_ wobble mutant. Five mutations were introduced into the Pam sequence and seed region of ACSL4_42-711_ in the pFLAG-CMV2-ACSL4_42-711_ wobble mutant by site-directed mutagenesis using a set of primers (forward, 5′-ATATTTGAAACATGTCTGTGCTGCAATCATCC-3′; reverse, 5′-ACATGTTTCAAATATAACTTTCCTCTTGTGAC-3′). Enzymatically defective ACSL4 was generated by following previously described methods (13). Briefly, G401R and G401L mutations were introduced into ACSL4_42-711_ of the pFLAG-CMV2-ACSL4_42-711_ wobble mutant by two sets of primers: 5′-CCGCATGATGCTGTCTAGAGGGGCCCCGCTATC-3′ and 5′-GATAGCGGGGCCCCTCTAGACAGCATCATGCGG-3′ for the G401R mutation and 5′-GTCCGCATGATGCTGTCTTTAGGGGCCCCGCTATCTC-3′ and 5′-GAGATAGCGGGGCCCCTAAAGACAGCATCATGCGGAC-3′ for the G401L mutation.

### Quantitative RT-PCR.

Total RNA from the indicated cells was extracted using TRIzol reagent (Invitrogen). One microgram of RNA was used as a template to synthesize cDNA with ReverTra Ace (TOYOBO, Osaka, Japan). The Roche LightCycler 480 System and KAPA SYBR FAST qPCR master mix (Kapa Biosystems, Wilmington, MA, USA) were employed for the quantitative detection of nucleic acids. To detect the enterovirus 5′ untranslated region by real-time PCR, a set of primers was designed (forward, 5′-CCCTGAATGCGGCTAATC-3′; reverse, 5′-ATTGTCACCATAAGCAGCCA-3′), and actin was used as an internal control (forward primer, 5′-GCTCGTCGTCGACAACGGCTC-3′; reverse primer, 5′-CAAACATGATCCTGGGTCATCTTCTC-3′). SARS-CoV-2 RNA was extracted using the LabTurbo viral minikit with the LabTurbo 48 compact system. The cDNAs were synthesized using the MMLV reverse transcription kit (Protech, Springwood, Australia). The primers and probes targeting the E or RdRp genes were based on recommendations by the Taiwan Center for Disease Control (CDC). Primer and probe sequences were the following: E gene of SARS-CoV-2 forward, 5′-ACAGGTACGTTAATAGTTAATAGCGT-3′; reverse, 5′-ATATTGCAGCAGTACGCACACA-3′; probe, 5′FAM-ACACTAGCCATCCTTACTGCGCTTCG-BBQ-3′; RdRp gene of SARS-CoV-2 forward, 5′-GTGARATGGTCATGTGTGGCGG-3′; reverse 5′-CARATGTTAAASACACTATTAGCATA-3′; probe 5′-FAM-CAGGTGGAACCTCATCAGGAGATGC-BBQ-3′. The Roche LightCycler 480 system and 2× qPCRBIO probe blue mix Lo-ROX (Kapa Biosystems) were employed for the quantitative detection of nucleic acids.

### Plaque assay and plaque reduction assay.

Cells were seeded at 5 × 10^5^ cells per well in a 6-well plate and incubated at 37°C for 24 h. The virus was diluted 10-fold in a serum-free medium and added to the cells. For the plaque reduction assay, cells were pretreated with different doses of inhibitors. After 1 h, the virus was diluted to 50 PFU in a serum-free medium and added to the cells. After 1 h of adsorption, cells were washed with PBS and supplemented with 2% FBS and 0.3% agarose culture medium. Cells were incubated at 37°C for 48 to 96 h and then fixed with 10% formaldehyde. For CoV-OC43, Vero E6 cells were infected and incubated at 33°C. The cells were stained with crystal violet and viral plaques were counted and calculated as number of PFU per milliliter.

### Fluorescence microscopy.

ACSL4^+/+^ or ACSL4^−/−^ cells were infected with CV-A6 at an MOI of 20. After 7 h postinfection, cells were washed with PBS and fixed with 4% formaldehyde for 10 min at room temperature. Cells were permeabilized and immunostained with anti-dsRNA, anti-ACSL4, and anti-CNX antibodies and then stained with Alexa Fluor 488 goat anti-mouse (A11008; Invitrogen), Alexa Fluor 594 goat anti-rabbit (A11012; Invitrogen), or Alexa Fluor 647 donkey anti-goat (A21447; Invitrogen) secondary antibodies. The nuclei were stained with 4′,6-diamidino-2-phenylindole (DAPI). The cells were examined under a confocal laser-scanning microscope (LSM780; Zeiss, Oberkochen, Germany).

LLC-MK2 cells were infected with SARS-CoV-2 at an MOI of 0.01. At 4 days postinfection, cells were washed with PBS and fixed with 4% formaldehyde for 10 min at room temperature. Cells were permeabilized and immunostained with anti-SARS-CoV-2 spike and anti-SARS-CoV-2 nucleocapsid antibodies and then stained with Alexa Fluor 488 goat anti-mouse (A11001; Invitrogen) or Alexa Fluor 594 goat anti-rabbit (A11012; Invitrogen) secondary antibodies. The nuclei were stained with DAPI. The cells were examined under a fluorescence microscope (Olympus IX71; Tokyo, Japan).

### Transmission electron microscopy.

Approximately 1.5 × 10^6^ ACSL4^+/+^ or ACSL4^−/−^ cells were infected with CV-A6 at an MOI of 20. After 7 and 9 h postinfection, mock- and virus-infected cells were fixed in a solution that contained 3% glutaraldehyde and 2% paraformaldehyde in 0.1 M cacodylate buffer (pH 7.4) for 2 h at 4°C. Cells were washed and postfixed in 1% osmium tetroxide for 1 h and then incubated in 4% uranyl acetate for 2 h at room temperature. Samples were dehydrated at 4°C in 0%, 50%, 70%, 95%, 95%, and 100% alcohol. After treatment with alcohol-Epon (1:1) for 7 h at room temperature, samples were embedded in 100% Epon resin. Polymerization of the samples was performed in an oven at 35°C for 6 h, 45°C for 6 h, and 60°C for 24 h. The embedded samples were sliced into sections of 80 nm (±5 nm) and poststained with 4% uranyl acetate in H_2_O and lead citrate. Images were obtained using a Hitachi HT7800 transmission electron microscope.

### GSH determination.

GSH levels were measured by the GSH/GSSG-Glo assay (Promega) according to the manufacturer’s instructions. Briefly, RD cells were seeded in a 96-well plate at 2,000 cells/well and then infected with CV-A6 at an MOI of 1 for 24 h. The medium was replaced with Hanks’ balanced salts before adding total glutathione lysis reagent or oxidized glutathione lysis reagent, and the plate was shaken for 5 min at room temperature. Subsequently, cells were supplemented with luciferin generation reagent and luciferin detection reagent to measure luminescence.

### Antibodies.

Anti-ACSL4 (A-5) (diluted 1:500; sc-271800) and anti-GPX4 (E-12) (diluted 1:500; sc-166570) antibodies were purchased from Santa Cruz Biotechnology (Dallas, TX, USA). Anti-dsRNA (diluted 1:1,000; J2) was purchased from Scicons (Szirák, Hungary). Anti-CA-A6 VP1 (diluted 1:2,000; GTX132346), anti-SARS-CoV-2 spike (diluted 1:1,000; GTX632604), anti-SARS-CoV-2 nucleocapsid (diluted 1:1,000; GTX135361), anti-influenza A virus M1 (diluted 1:1,000; GTX125928), anti-ZIKA virus NS2B (diluted 1:1,000; GTX133308), and anti-ZIKA virus NS3 (diluted 1:1,000; GTX133309) antibodies were obtained from GeneTex (Irvine, CA, USA). Anti-caspase-1 (diluted 1:1,000; MAB6215) and anti-human RIPK3/RIP3 (diluted 1 μg/ml; MAB7604) were purchased from R&D Systems (Minneapolis, MN, USA). Anti-calnexin (diluted 1:500; ab219644) and anti-RIP3 (phospho-S227) (diluted 1:2,000; ab209384) were purchased from Abcam (Cambridge, UK). Anti-PARP (diluted 1:1,000; number 9542), anti-cleaved PARP (Asp214) (diluted 1:1,000; number 9541), anti-MLKL (D2I6N) (diluted 1:1,000; number 14993), anti-phospho-MLKL (Ser358) (D6H3V) (diluted 1:1,000; number 91689), and anti-gasdermin D (E8G3F) (diluted 1:1,000; number 97558) were obtained from Cell Signaling (Danvers, MA, USA). Anti-actin (diluted 1:4,000; MAB1501) antibody was purchased from Merck Millipore (Burlington, MA, USA). Glyceraldehyde-3-phosphate dehydrogenase (GAPDH) (diluted 1:2,000; H00002597-M01) was purchased from Abnova (Taipei, Taiwan).

### Chemicals.

Ferrastatin-1 (SML0583), troglitazone (T2573), rosiglitazone (R2408), pioglitazone (E6910), RSL3 (SML2234), glutathione reduced ethyl ester (G1404), Q-VD-OPh (SML0063), necrostatin-1 (N9037), and disulfiram (D2950000) were purchased from Merck.

### Statistical analysis.

Experimental data were analyzed using Student’s two-tailed unpaired *t* tests and two-way analysis of variance (ANOVA) using GraphPad Prism 8. *P *values of *<*0.05 were statistically significant.
