# Supplementary figures and images for: Acyl-Coenzyme A Synthetase Long-Chain Family Member 4 Is Involved in Viral Replication Organelle Formation and Facilitates Virus Replication via Ferroptosis
Source: mBio. 2022 Jan 18;13(1):e02717-21. doi: 10.1128/mbio.02717-21 (PMC8764547; doi:10.1128/mbio.02717-21)

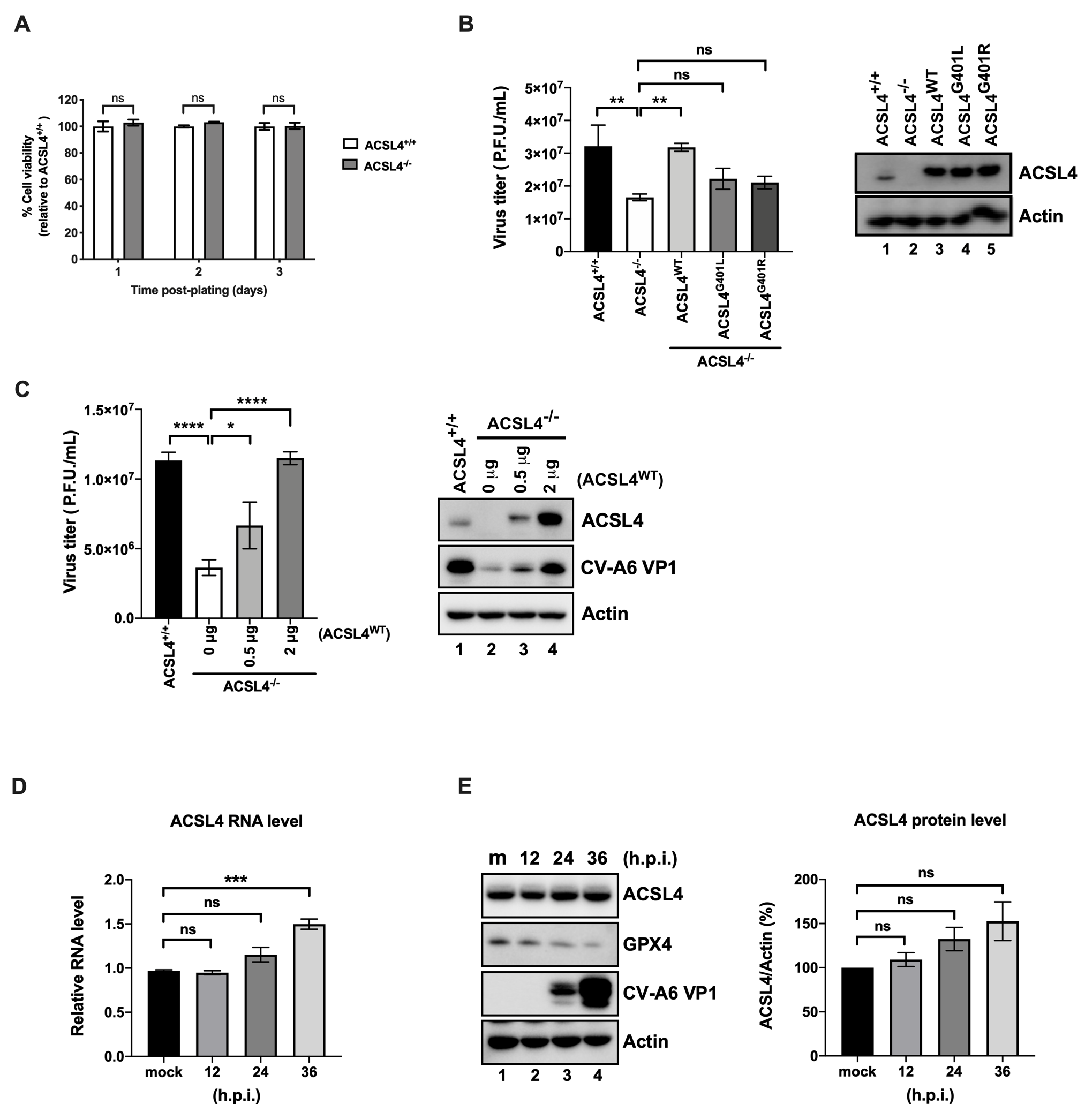

Supplement: FIG S1 [file mbio.02717-21-sf001.tif]

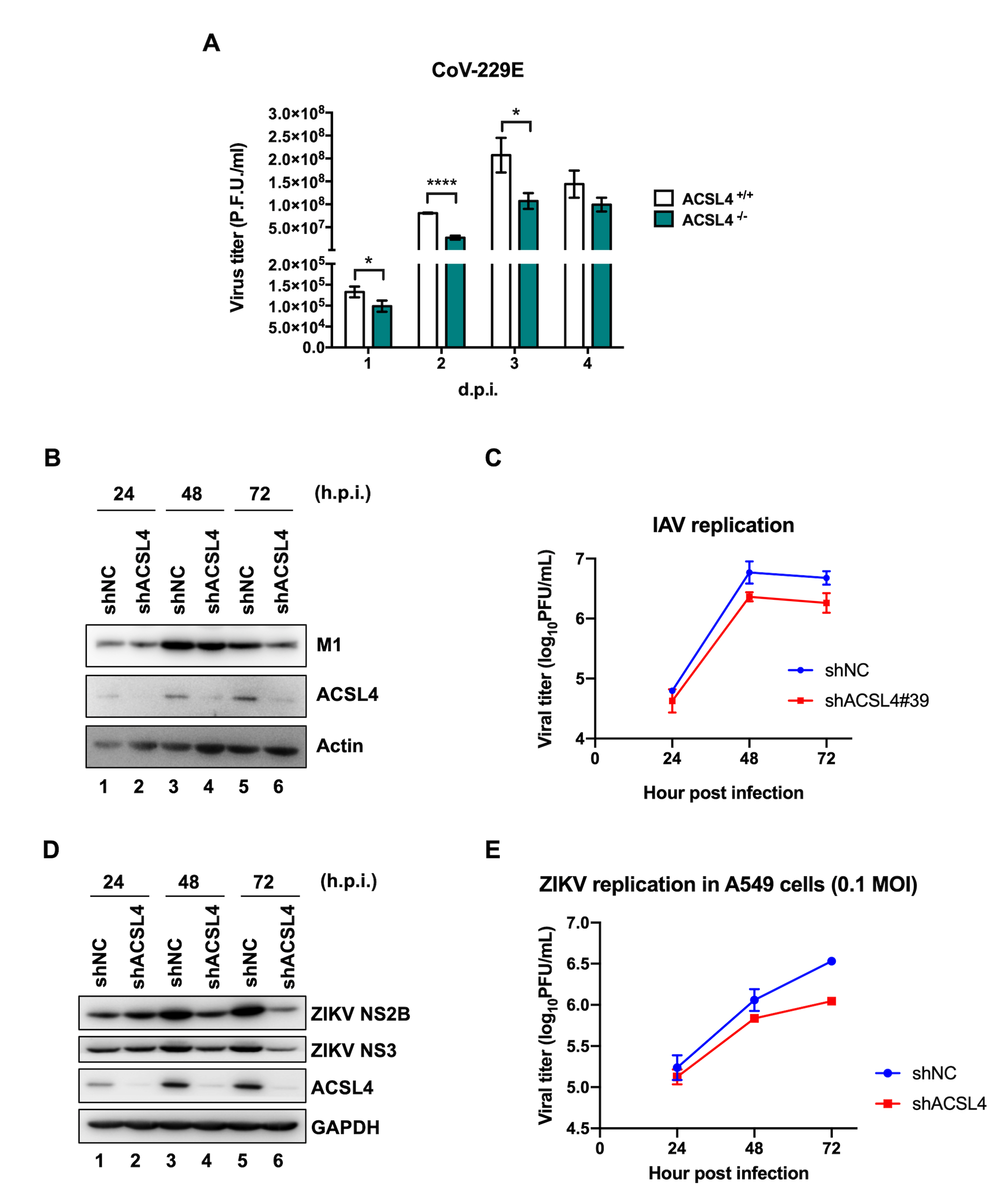

Supplement: FIG S2 [file mbio.02717-21-sf002.tif]

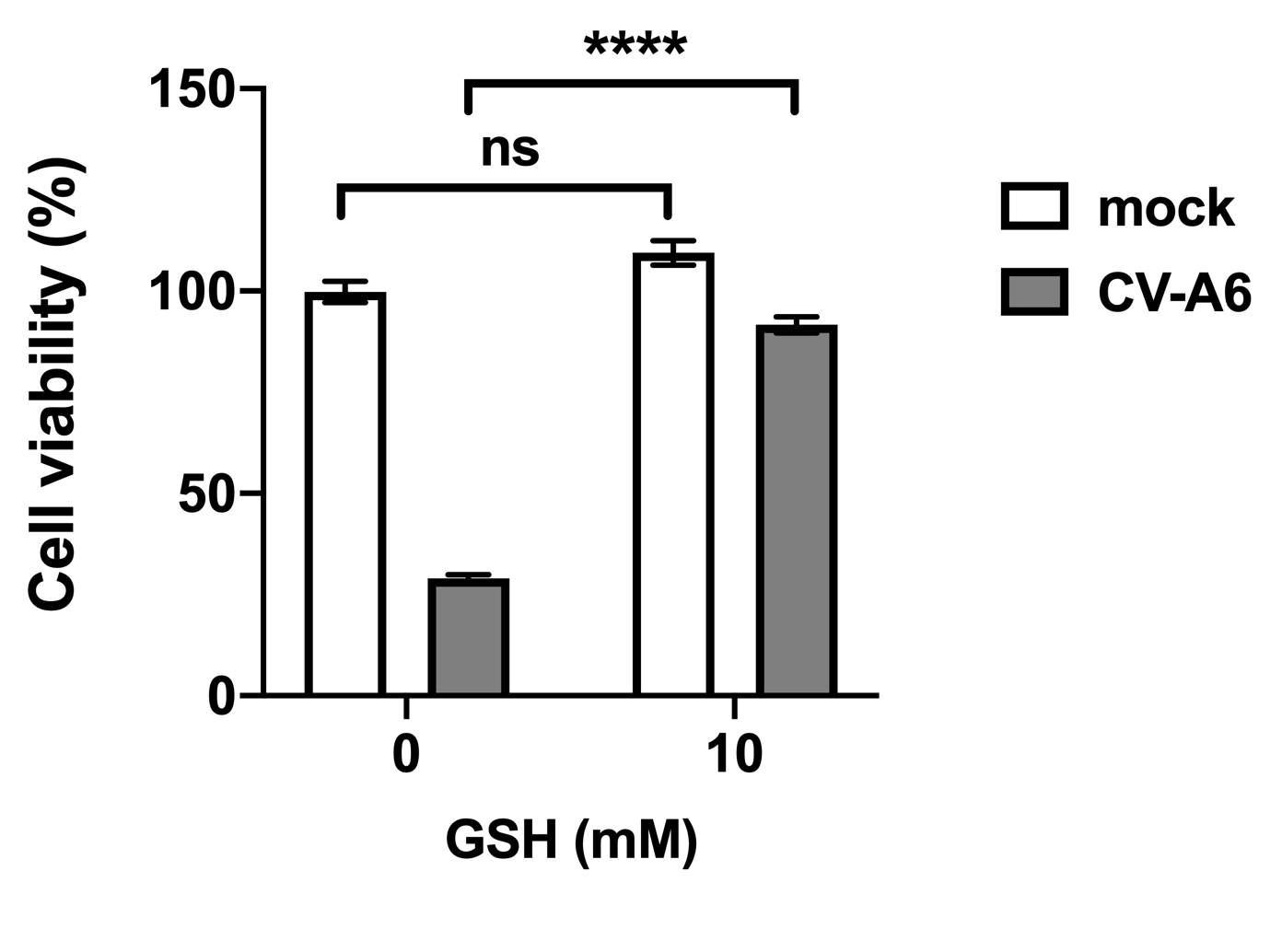

Supplement: FIG S3 [file mbio.02717-21-sf003.tif]

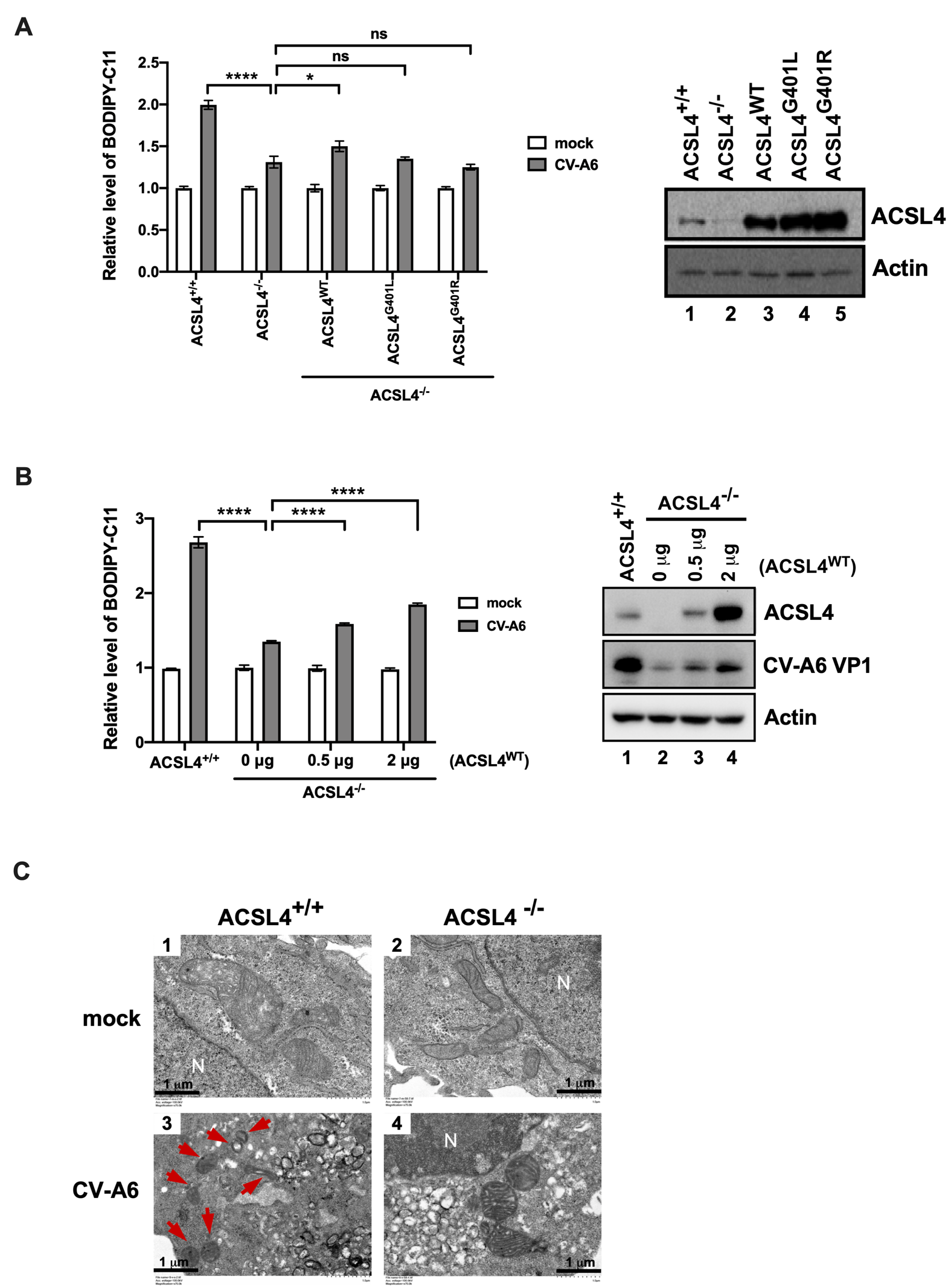

Supplement: FIG S4 [file mbio.02717-21-sf004.tif]

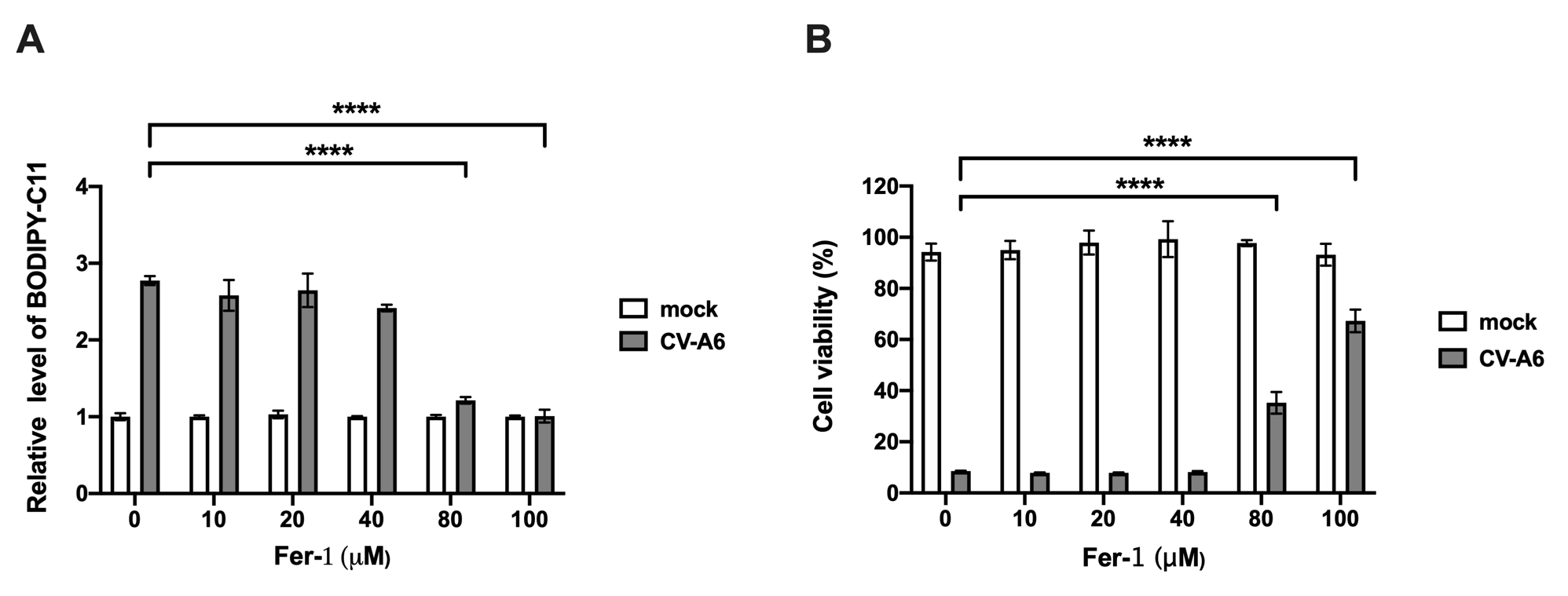

Supplement: FIG S5 [file mbio.02717-21-sf005.tif]

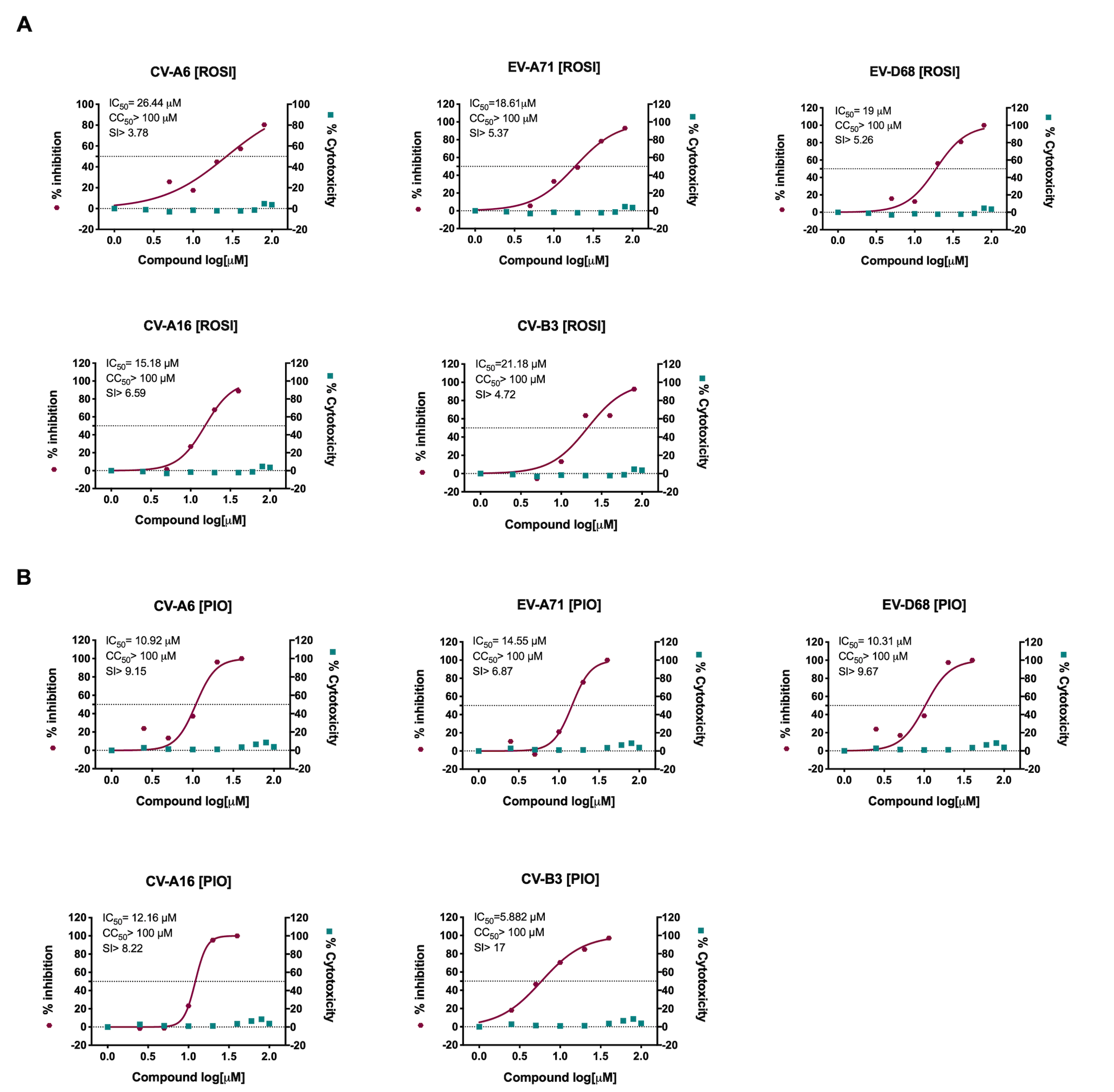

Supplement: FIG S6 [file mbio.02717-21-sf006.tif]

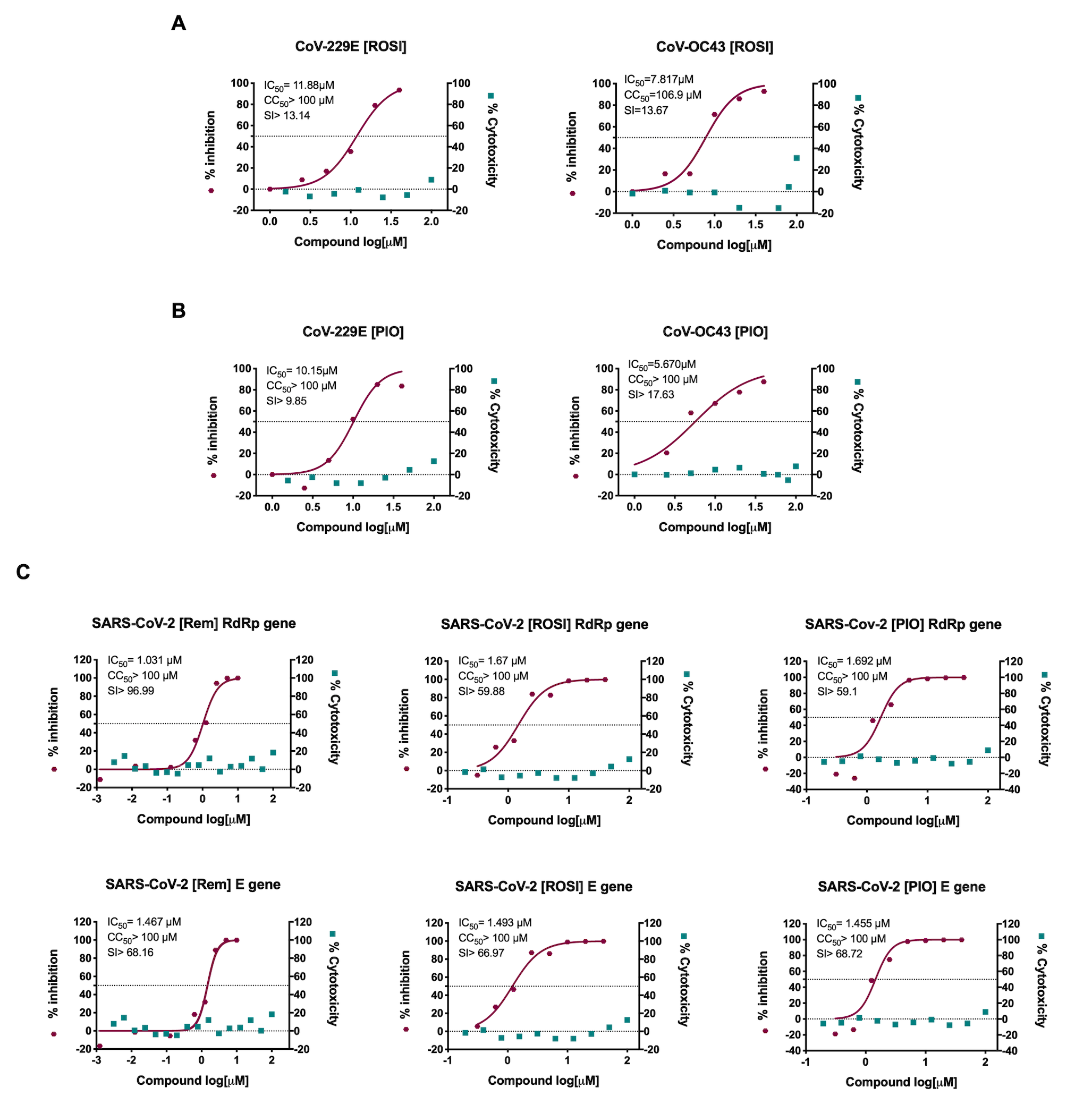

Supplement: FIG S7 [file mbio.02717-21-sf007.tif]

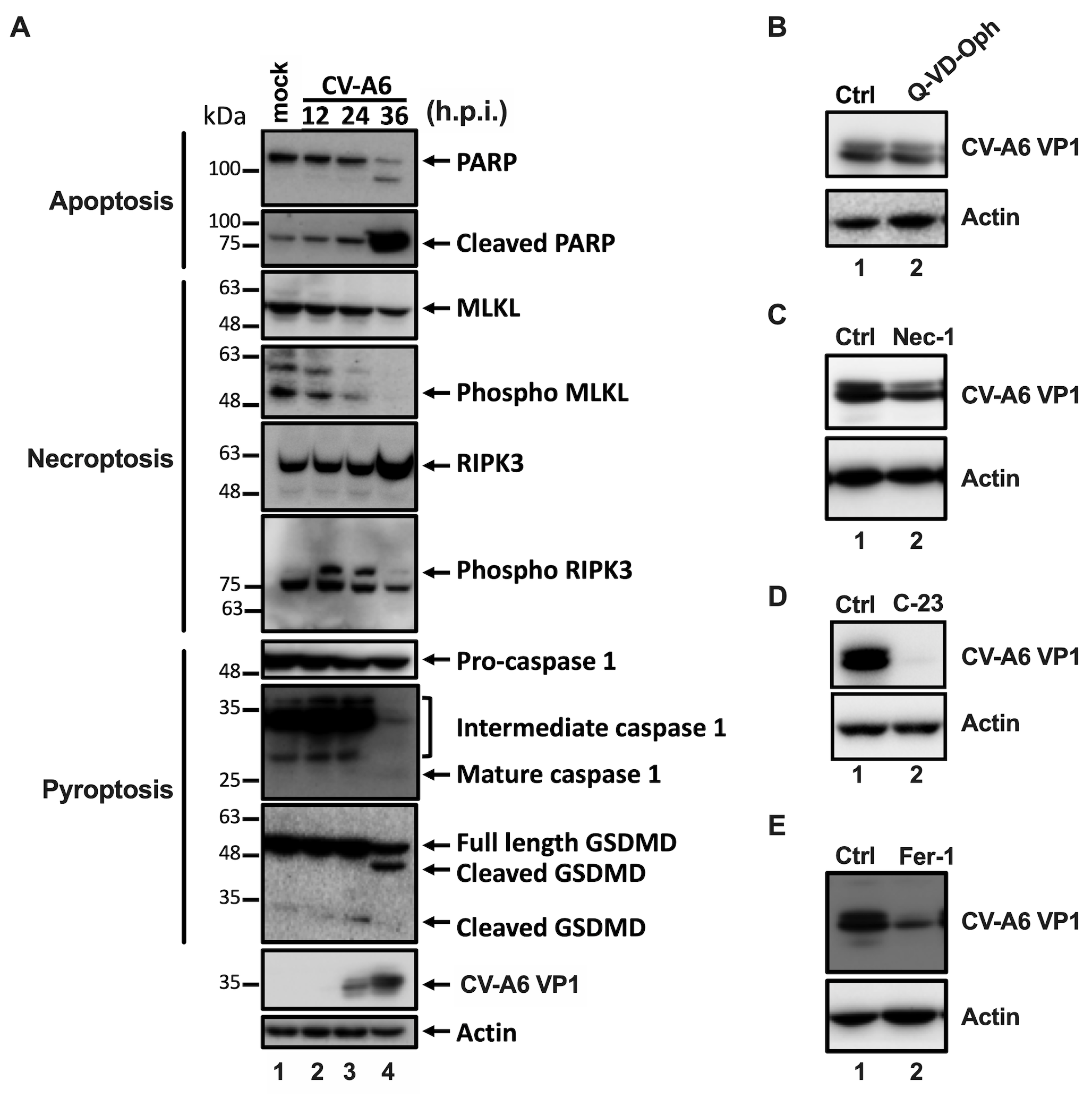

Supplement: FIG S8 [file mbio.02717-21-sf008.tif]
